# Crosstalk in concurrent repeated games impedes direct reciprocity and requires stronger levels of forgiveness

**DOI:** 10.1038/s41467-017-02721-8

**Published:** 2018-02-07

**Authors:** Johannes G. Reiter, Christian Hilbe, David G. Rand, Krishnendu Chatterjee, Martin A. Nowak

**Affiliations:** 1 Program for Evolutionary Dynamics, Harvard University, Cambridge, MA 02138 USA; 2 IST Austria (Institute of Science and Technology Austria), Klosterneuburg, 3400 Austria; 3 Yale Institute for Network Science and Department of Psychology and Department of Economics, Yale University, New Haven, CT 06520 USA; 4 Department of Organismic and Evolutionary Biology and Department of Mathematics, Harvard University, Cambridge, MA 02138 USA; 5 Present Address: Canary Center for Cancer Early Detection, Department of Radiology, Stanford University School of Medicine, Palo Alto, CA 94304 USA

## Abstract

Direct reciprocity is a mechanism for cooperation among humans. Many of our daily interactions are repeated. We interact repeatedly with our family, friends, colleagues, members of the local and even global community. In the theory of repeated games, it is a tacit assumption that the various games that a person plays simultaneously have no effect on each other. Here we introduce a general framework that allows us to analyze “crosstalk” between a player’s concurrent games. In the presence of crosstalk, the action a person experiences in one game can alter the person’s decision in another. We find that crosstalk impedes the maintenance of cooperation and requires stronger levels of forgiveness. The magnitude of the effect depends on the population structure. In more densely connected social groups, crosstalk has a stronger effect. A harsh retaliator, such as Tit-for-Tat, is unable to counteract crosstalk. The crosstalk framework provides a unified interpretation of direct and upstream reciprocity in the context of repeated games.

## Abstract (web summary)

Social interactions among people are often repeated, and yet it is assumed that simultaneous interactions are independent from one another. Here, Reiter and colleagues describe a conceptual framework where an action in one game can influence the decision in another.

## Introduction

Social dilemmas are situations where mutual cooperation is better than mutual defection and yet there is an incentive to defect^1,2^. Cooperation is normally opposed by natural selection unless mechanisms for the evolution of cooperation are in place^3^. One such mechanism is direct reciprocity, which is based on repeated interactions between the same two players^4,5^. In repeated social dilemmas, humans often learn to use adaptive rules, telling them when to cooperate, when to defect, and how to motivate others to cooperate^6–8^. Cooperation can be achieved if people adopt conditional cooperative strategies such as Tit-for-Tat^5^, Generous Tit-for-Tat^9,10^, or Win-stay, Lose-shift^11,12^. Conditional cooperation, paired with some amount of generosity, can maintain a healthy level of cooperation^13–21^. It can evolve even if initially rare^22–26^.

**Fig. 1:**
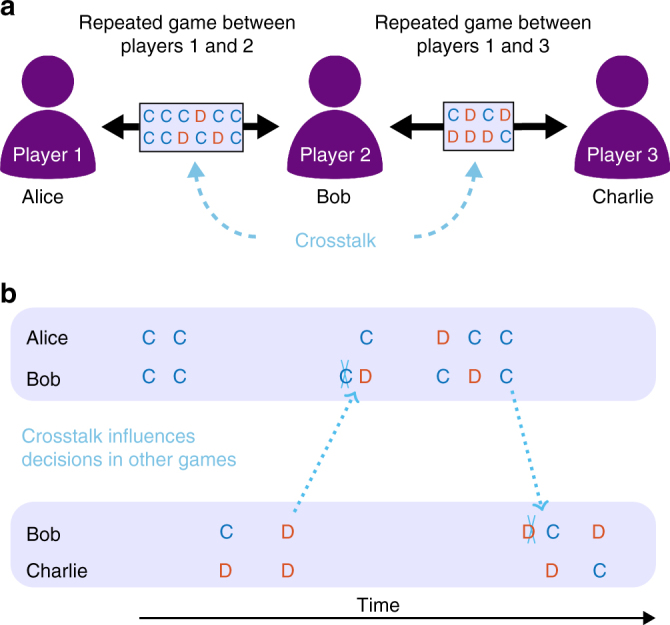
Crosstalk between repeated games. **a** Bob is engaged simultaneously in independent, repeated games with Alice and Charlie. Crosstalk means that the moves that occur between Alice and Bob can affect Bob’s decisions towards Charlie. **b** Alice and Bob as well as Bob and Charlie simultaneously play a repeated Prisoner’s Dilemma. The rounds of the two games occur at particular times. In each round the players can either cooperate (C) or defect (D). The outcome of a round in one game can influence the subsequent round in the other game. Crosstalk is indicated by dotted arrows

Most previous models of direct reciprocity (with a few notable exceptions^27–30^) have either assumed that (i) individuals only engage in one repeated game at a time or that (ii) an individual’s action in one game is independent of all its other interactions. Because humans often engage in many games simultaneously, the first assumption seems to be violated in most practical scenarios. Moreover, evidence from experimental studies suggests that also the second assumption of independence may not always apply^31–38^. We say that a player’s decision is subject to “crosstalk” when an interaction that a player has in one repeated game influences how the very same player behaves in another repeated game (Fig. 1a). For example, consider the interactions in a group of three individuals, “Alice”, “Bob”, and “Charlie” (Fig. 1b). Suppose that after a series of previous encounters, Bob is prompted for a decision whether to cooperate with Alice in the next round. In her last interaction with Bob, Alice has cooperated. Therefore, Bob who uses Tit-for-Tat, would now cooperate with Alice. But Bob’s last interaction had occurred with Charlie and in that interaction Charlie had defected. Crosstalk now means there is some chance that Bob defects with Alice although direct reciprocity would mandate Bob to cooperate. Bob’s state with respect to Charlie influences his decision with respect to Alice.

Such crosstalk can result from various psychological processes. For example, experiments on upstream reciprocity suggest that subjects who have received help in their previous interaction often consciously choose to “pay it forward”^22,31–33^. Alternatively, crosstalk may also occur when subjects have limited working memory^34–36^. In that case, subjects may confuse their co-players’ past actions, which may in turn lead them to reward the wrong person for past cooperative behaviors. We propose a mathematical framework that allows us to quantify how crosstalk affects the cooperation dynamics within a population. We show that, in the presence of crosstalk, a single defector can lead to the complete breakdown of cooperation in an arbitrarily large group of conditional cooperators. Nevertheless, cooperation can prevail if the population is structured and if subjects are sufficiently forgiving. For our model, we do not need to specify the particular psychological process at work: the resulting behavioral dynamics are independent of whether crosstalk is the result of a conscious decision (as in upstream reciprocity), or the consequence of a subconscious error (as when individuals confuse the past actions of their co-players). However, the interpretation of our results will often depend on the specific psychological mechanism that gives rise to crosstalk. We revisit this matter in the “Discussion” section.

## Results

### Framework for crosstalk between concurrent repeated games

We consider a group of *N* individuals. Each individual plays a pairwise repeated prisoner’s dilemma (PD) with each interaction partner. These repeated games occur concurrently. At each time step, we choose a random pair of players for a single interaction (Fig. 1b). Each player uses a reactive strategy, defined by two parameters, *p* and *q*, which denote the probability to cooperate if the same co-player in the previous round has either cooperated or defected, respectively. The class of reactive strategies includes many well-known examples, such as always-cooperate (ALLC), always-defect (ALLD), tit-for-tat (TFT: Supplementary Fig. 1b), and Generous Tit-for-Tat^10^ (GTFT: Supplementary Fig. 1d). Reactive strategies can be implemented by stochastic two-state automata^39–42^. The two states are labeled C and D (see Supplementary Fig. 1a). In the next interaction, a player cooperates if she is in state C and defects if she is in state D. Cooperators pay a cost *c* for their co-player to receive a benefit *b>c*. Defectors do not incur a cost and their co-player does not receive a benefit. The player’s strategy determines how the player’s state is updated after an interaction has taken place.

In our setup, each player uses a specific strategy for all of her interactions, but has distinct automata to hold the games with all of her different co-players in memory (Supplementary Fig. 2). For example, a player using TFT can be in different states (C or D) with different co-players, but uses the same strategy to update her states against all of her co-players. The separate automata enable players to remember previous interactions and to react in future rounds according to their respective history with each co-player.

**Fig. 2:**
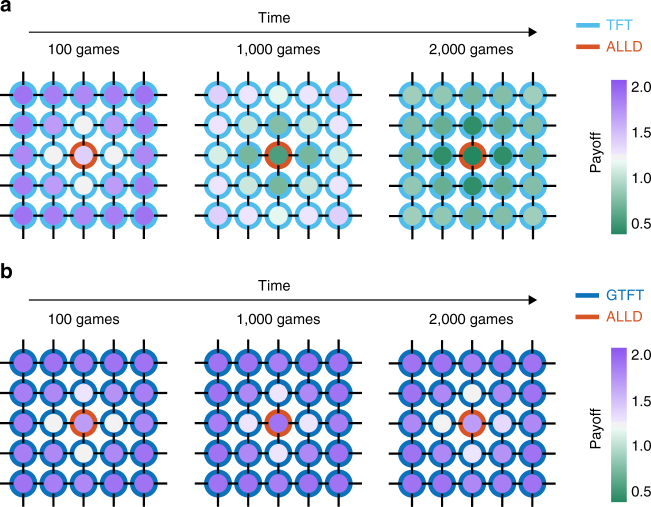
Cooperative and defective behavior can spread across a population in the presence of crosstalk. Twenty-four conditional cooperators (blue framed nodes, panels) and one ALLD (Always-Defect) player (red framed node, placed in the center) populate a 5 × 5 lattice. The fill color of the nodes depicts the expected payoff of the players after 100, 1000, and 2000 games. **a** If the conditional cooperators use TFT (Tit-for-Tat), crosstalk leads to the spread of defection from the ALLD player to all other group members. Cooperation goes extinct. **b** GTFT (Generous Tit-for-Tat) is an error-correcting strategy and can thereby suppress the spread of defection by crosstalk. Only the players in the neighborhood of the ALLD player have reduced payoff. Parameter values: crosstalk rate *γ* = 0.5, benefit *b* = 3, and cost *c* = 1. For GTFT (defined by *p* = 1 and 0 < *q* < 1), we used *q* = 1/3

**Fig. 3:**
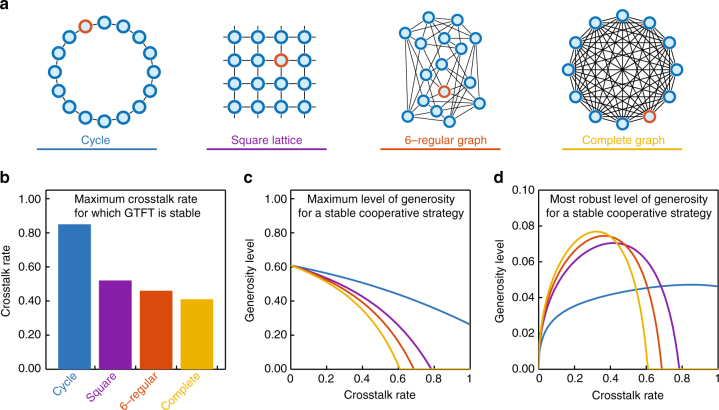
Population structure and crosstalk rate substantially affect stationary payoffs and evolutionary stability. **a** Examples of the investigated population structures: cycle, square lattice, 6-regular graph, complete graph. Defectors (red nodes) are randomly placed within the population. **b** Maximally tolerated crosstalk rate (*γ*) in various population structures such that the payoff of the ALLD player remains below the payoff of the GTFT players (for a generosity parameter *q* fixed to 1/3). **c** Maximum level of generosity (*q*_M_) such that the average payoff of the GTFT players exceeds the payoff of the ALLD player. The circle allows players to be most generous when crosstalk is common. **d** Most robust level of generosity (*q*_R_) maximizing the relative payoff advantage of the GTFT players compared to the ALLD player. When crosstalk is rare, a small increase in the crosstalk rate allows players to be more generous, to decrease the chance that defection spreads across the population. However, when crosstalk becomes common, a further increase of *γ* requires the players to become less generous to constrain the payoff of the ALLD player. Parameter values: number of players *N* = 16 (one ALLD player), benefit *b* = 3, and cost *c* = 1

Crosstalk between two repeated games occurs if a player’s state with respect to one interaction partner displaces the player’s state with respect to another player (Supplementary Fig. 2). Specifically, we assume that, before each interaction, there is a probability *γ* that the players’ state with respect to the current co-player is replaced by the state with respect to the previous co-player (other variants of crosstalk will be discussed below). The crosstalk rate *γ* ∈ [0, 1] specifies how often crosstalk occurs. In the special case of no crosstalk, *γ* = 0, players perfectly distinguish between all their opponents, and we recover the scenario considered in previous studies of direct reciprocity^2,5^. For positive crosstalk rates, cooperative and defective behaviors can cascade in the players’ social network: a player’s action in one game can affect how the co-player acts in a different game, which in turn may influence again other games (Fig. 1). Therefore, crosstalk causes ripples that propagate in social networks. The overall effect of crosstalk depends on the structure of the population. We represent this structure by arranging players on a graph^43–45^, where edges between players denote interactions (Fig. 2; “Methods” section). While our framework is applicable to arbitrary population structures, we illustrate the effects of crosstalk using four regular networks (Fig. 3a), ranging from a circle (where each player has exactly two interaction partners) to the complete graph (where all players interact with everyone else).

### Cooperative and defective behavior spreads across population

We utilize stochastic computer simulations to study the cooperation frequency in a population over time, and derive mathematical recursions to calculate the long-run payoffs in the steady state (“Methods” section). To illustrate how crosstalk leads to the spread of defection in a generally cooperative society, we place a single ALLD player in a network of *N* − 1 conditionally cooperative players (Figs. 2 and 3a). When the conditionally cooperative players use TFT (which is given by *p* = 1, *q* = 0) and crosstalk occurs, *γ* > 0, the ALLD player can turn all remaining players into defectors eventually (Fig. 2a), independent of the population structure and the crosstalk rate (Supplementary Fig. 3). The spread of defection can be prevented if the cooperative players use more generous strategies, with *p* = 1 and 0 < *q* < 1. We refer to such strategies as GTFT. The impact of different *q* values will be discussed below. At first, we choose *q* = 1/3. If the single ALLD player is placed among GTFT players, cooperation frequencies converge to a positive value (Fig. 2b), with the eventual equilibrium rates depending on the population structure and on the crosstalk rate (Supplementary Fig. 3). We can also observe the opposite effect: a single ALLC player can increase the cooperation rates in a population of stochastic TFT players using *p* = 1 − $\epsilon$ and *q* = $\epsilon$ (Supplementary Fig. 4c).

Comparing the effect of different population structures, we find that a GTFT population can maintain cooperation more easily if players are arranged on a circle instead of a complete graph (Fig. 3a, b, Supplementary Fig. 5). For a population size of *N* = 16, the cooperators obtain a higher average payoff than the defector for crosstalk rates up to *γ* = 0.85 on a circle, and for up to *γ* = 0.41 on a complete graph. In networks with a low degree, players are more likely to give the adequate response with respect to their current co-player, because, if crosstalk occurs, the current co-player is more likely to coincide with the previous co-player such that crosstalk becomes inconsequential. For this reason, all other explored population structures exhibit crosstalk thresholds between the circle and the complete graph (Fig. 3b).

To investigate the recovery properties after a mistake, we computed the amount of time that a population of conditional cooperators with strategy (1, *q*) and *q* > 0 needs to return to full cooperation after a single defection event. We find that crosstalk leads to a significantly faster recovery (Supplementary Fig. 6). Intuitively, when the crosstalk rate is high, a player’s automata are updated more frequently (once before the interaction takes place, and once after the interaction). Because the players apply strategies with *p* = 1 and *q* > 0, each updating event is biased towards increasing cooperation: every cooperative act puts the co-player into a cooperative state, whereas defective acts are forgiven with probability *q*. Moreover, we show that the recovery time is monotonically increasing with the average degree (*k*) of the population structure and monotonically decreasing with the probability to cooperate after defection (*q*; see Supplementary Note 1 for more details).

### Crosstalk requires the right level of forgiveness

We calculate the generosity parameter *q* of GTFT to optimally cope with crosstalk. To this end, we consider two different optimality criteria. First, we calculate the most generous strategy that is able to resist invasion by a single ALLD player. That is, for a fixed population structure and a given crosstalk rate, we derive the reactive strategy (1, *q*_M_) with maximum *q*_M_ such that *N* − 1 players with this strategy get at least the same average payoff as the single defector. Analytical calculations for the complete graph and numerical results for all other population structures show that higher crosstalk rates and higher network degrees (that is a higher number of neighbors) require the cooperative players to be less generous (Fig. 3c, “Methods” section). For the second optimality criterion, we calculate the cooperative strategy with the most robust level of generosity *q*_R_ such that *N* − 1 players with strategy (1, *q*_R_) have the highest relative payoff advantage compared to the single ALLD player. The most robust level of generosity exhibits a non-monotonic dependence on the crosstalk rate (Fig. 3d). In the absence of crosstalk, *γ* = 0, the perfectly reciprocal TFT strategy is most robust against invasion of ALLD. As the crosstalk rate increases, the most robust level of generosity *q*_R_ first increases, but then decreases again. Intuitively, for the robustness of a conditionally cooperative population against ALLD, high values of the generosity parameter *q* have two opposing effects. On one hand, high values of *q* make it less likely that the defectors’ actions propagate through the network. On the other hand, high values of *q* also let the players be more forgiving against the defector, and hence increase the payoff of the ALLD player. When crosstalk is rare, conditional cooperators can prevent the spread of defection by choosing a small value for *q*. Once crosstalk is sufficiently frequent, however, players can no longer fully prevent defection from spreading. Instead, they rather need to keep the defector’s payoff low, by choosing a smaller *q* value. These results confirm that in the presence of crosstalk, *γ* > 0, subjects should show some amount of generosity (*q* > 0), but not too much; we find *q* < *q*_M_, with *q*_M_ depending on the crosstalk rate and on the population structure. Only for the circle, cooperation can prevail even when crosstalk is abundant (Fig. 3d).

The above analysis is based on a comparison between conditional cooperators and a specific invader, ALLD. More generally, we find that conditionally cooperative strategies (1, *q*) with *q* < *q*_M_ in fact resist invasion by all possible invading strategies (*p*ʹ, *q*ʹ) for the complete graph. This analysis also reveals that there are three classes of strategies in total that are stable against arbitrary invaders (Supplementary Note 1). The first class consists of the conditionally cooperative strategies just described. The second class consists of uncooperative strategies (*p*, 0), with *p* sufficiently small (see Supplementary Note 1 for the exact condition). In particular, this class contains ALLD. When adopted by all players in a population, strategies of this class eventually lead to full defection. Finally, the third class consists of strategies (*p*, *q*) analogous to equalizer strategies of direct reciprocity^46–49^. When applied by all residents in the population, equalizer strategies guarantee that the payoff of a single invader is independent of the invader’s strategy.

### Crosstalk impedes the evolution of cooperation

**Fig. 4:**
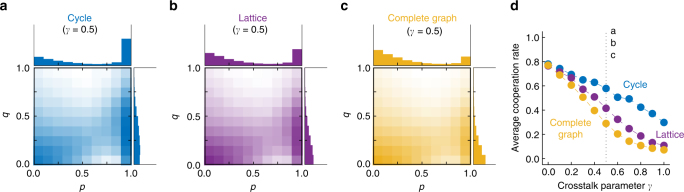
Cooperative strategies evolve for various crosstalk rates and population structures. **a**–**c** Abundance of different reactive strategies (*p*, *q*) over the course of an evolutionary process when the crosstalk rate is fixed to *γ* = 0.5. The probability to cooperate if the co-player in the previous round either cooperated or defected is denoted by *p* and *q*. On a cycle and a lattice, GTFT strategies evolved, whereas the same settings did not allow for the evolution of cooperative strategies on a complete graph. **d** Resulting average cooperation rates across different crosstalk rates. The dotted line indicates the crosstalk rate used in **a**–**c**. See “Methods” section for setup of the simulations. Parameter values: population size *N* = 16, benefit *b* = 10, cost *c* = 1, intermediate selection strength *s* = 1

These results raise the question to which extent subjects themselves would learn to apply cooperative strategies with a sustainable degree of generosity. To explore that question, we implemented a simple model of cultural evolution where players are allowed to adopt new strategies over time, based on their current strategy’s success (“Methods” section). According to this process, strategies that yield a comparably high payoff are more likely to be imitated by other players^50,51^. In addition, players may occasionally also experiment with new stochastic strategies, which introduces novel behaviors into the population. These two events, imitation and exploration, take the role of selection and mutation in models of biological evolution. We show that a birth-death process as used in many biological applications yields the same results (Supplementary Note 1). We simulated the evolutionary dynamics for various population structures and crosstalk rates, assuming that experimentation events are relatively rare^23^. In the plane of reactive strategies, we observe that for most of the time, players either apply defective strategies with *p* ≈ *q* ≈ 0, or cooperative strategies with *p* ≈ 1 and 0 < *q* < *q*_M_ (Fig. 4a–c). None of these strategies are evolutionarily stable^52^. Instead, when residents apply one of these strategies, neutral or nearly neutral mutants can often invade and pave the way for mutants of another strategy class^42^. The relative weight of these two strategy classes depends on the crosstalk rate and on the population structure (Fig. 4d). While cooperative strategies readily evolve on the cycle even for substantial crosstalk rates, they become less abundant as the population structure changes to a square lattice, or to a complete graph.

To understand the effect of crosstalk in more detail, we analyzed how easily other strategies fix in a resident population that either consists of ALLD players or GTFT players (Supplementary Fig. 7). Without crosstalk, GTFT is much more successful in resisting mutant invasions. On average, more mutants need to be introduced until the first mutant fixes. Moreover, successful mutants typically have a strategy that is very similar to GTFT (whereas ALLD is typically invaded by TFT-like strategies rather quickly). However, as the crosstalk rate increases, the invasion time into GTFT drops considerably, and successful mutants no longer need to be cooperative themselves.

Interestingly, we find that crosstalk favors the stability of extortionate strategies. With an extortionate strategy, players can guarantee that they never get a lower payoff than their co-player, while simultaneously acting such that it is in the co-player’s best interest to cooperate unconditionally^47^. In classical models of direct reciprocity, extortionate strategies are unstable and they can only succeed if the population size is small^18,19,53^. In contrast, extortionate strategies can thrive even in large populations when crosstalk is sufficiently abundant (Supplementary Fig. 8). However, this success comes at a cost. By becoming stable, extortionate strategies lose one of their most appealing properties: when crosstalk is abundant, a rare invader in an extortionate population does no longer benefit from being unconditionally cooperative. Instead, the best response is to be extortionate as well (see Supplementary Note 1 for details).

To analytically understand the impact of crosstalk on the evolutionary dynamics, we explored a deterministic model of evolution in well-mixed populations^54–56^. The singular strategies of these dynamics consist exactly of the three strategy sets that resist invasion by rare mutants that we described in the previous section: conditional cooperators, defectors, and equalizers. Which of these strategy classes is reached in the course of evolution now depends on the initial population (Supplementary Fig. 9a). As the crosstalk rate increases, the number of initial populations that eventually end up in a conditionally cooperative state decreases (Supplementary Fig. 9b). In this deterministic model, crosstalk thus acts by reducing the basin of attraction of the cooperative equilibria.

### Alternative models of crosstalk

So far, we have analyzed one particular model of crosstalk: when prompted for the next decision, a player instead reacts to her most recent interaction, and this interaction could have been with someone else. But other implementations are conceivable.

For example, the memory state that a player holds for her current co-player could be replaced by the memory state of a random co-player, who may not coincide with the current or the most recent co-player. In this case, when deciding what to do for the next round in a particular game, the player uses with probability *γ* the state of a random game that she is holding in memory. We find that this alternative implementation of crosstalk differs in its short-term dynamics but converges to the same steady state as our original model (Supplementary Fig. 10). Thus, Figs. 3 and 4 immediately apply to this type of crosstalk as well.

Alternatively, a player’s decision could depend not only on the previous interaction with one particular opponent. Instead, the player might consider an average across her recent experiences with all her co-players. We therefore introduce aggregate reactive strategies (Supplementary Note 1). Players using these strategies compute a weighted cooperation score across all their co-players. This cooperation score incorporates the last action of the current co-player with weight 1 − *γ* and the average cooperation rate across all co-players’ last actions with weight *γ*. The obtained score is then compared to an exogenous cooperation threshold *τ*. Players cooperate with probability *p* if the weighted cooperation score exceeds *τ*; otherwise they cooperate with probability *q*. In particular, if the crosstalk rate *γ* is zero, these strategies again correspond to the classical reactive strategies of direct reciprocity^2,10^. We explore the effects of *γ* and *τ* by considering a single defector in a population of conditional cooperators. The conditional cooperators apply a strategy with *p* = 1, *q* = 1/3 and some value *τ*, a strategy to which we refer to as Aggregate Generous Tit-for-Tat (AGTFT). While the cooperation dynamics of this model are qualitatively different from our previous results, we again find that higher crosstalk rates impede the stability of cooperation across all population structures (Supplementary Figs. 11 and 12).

## Discussion

Classical models of direct reciprocity require that players provide a targeted response to each of their co-players^2,5^. Experimental results and everyday experience indicate that players’ decisions can be affected by unrelated events that occur in their interactions with others^31–38^. Crosstalk arises when a player has simultaneous repeated interactions with several opponents. Importantly, crosstalk is different from previous approaches that combined direct and indirect (downstream) reciprocity. In models of downstream reciprocity, a player’s strategy depends on the co-player’s reputation, and hence on the co-players’ interactions with others^38,57–64^. A combination of direct reciprocity and downstream reciprocity can promote cooperation because a single defection in one game may lead several unaffected co-players to retaliate against the defector^27–29^. However, downstream reciprocity makes stronger assumptions on the information players have when making their decision. It requires a player to observe other players’ interactions or reputations to respond accordingly. In contrast, crosstalk is a much more elementary mechanism. It occurs “within” each player and does not rely on additional external information about independent interactions of unrelated players. Our notion of crosstalk is general: it captures that a player’s decision in one game can be affected by the player’s previous experience in another game, but it does not depend on the psychological process responsible for this interdependency.

Depending on the specific process at work, crosstalk is amendable to different interpretations. Our framework can be taken as a model of upstream reciprocity in the context of repeated games. Under this interpretation, cooperation or defection received from one person is sometimes consciously “paid forward” to another person. Previous analytical models have either focused on direct reciprocity or on upstream reciprocity separately^2,65,66^. The framework of crosstalk allows us to explore the consequences when both modes of reciprocity act simultaneously, and possibly interfere with each other. We recover previous results^65–67^ that upstream reciprocity alone is most likely to yield cooperation when the population is highly structured. However, our results suggest that cooperation can even be maintained in well-mixed populations when upstream reciprocity is sufficiently coupled with direct reciprocity (i.e., when the crosstalk rate *γ* is sufficiently small).

Alternatively, crosstalk can serve as a model of individuals with limited working memory. According to this interpretation, crosstalk occurs when individuals confuse their various co-players, which introduces a type of behavioral noise into the cooperation dynamics. This noise is different from simple implementation errors considered in previous models^2,9,18,19,36,68,69^. Implementation errors only affect the repeated game in which they occur (Supplementary Fig. 4a, b). But crosstalk spreads from one game to another and therefore through the population (Supplementary Fig. 4). Only in the presence of crosstalk, a single defector can turn a whole population of TFT players into defectors.

We consider upstream reciprocity and confusion as different psychological processes which independently can give rise to crosstalk. Although these two processes are subject to different interpretations, according to the above discussed implementation, they lead to the same cooperation dynamics within a population. High degrees of upstream reciprocity, just as high degrees of confusion, undermine the ability of direct reciprocity to sustain cooperation.

Crosstalk provides a general framework with applications beyond the examples studied here. Future models could explore, for example, crosstalk between independent games that differ in their payoff structure^70^, or when subjects engage in simultaneous games that involve more than two players. Similarly, one may study interactions in which the crosstalk rate itself depends on exogenous parameters, such as the number of neighbors, or the benefit of cooperation. Finally, we explored a model in which players aggregate across their last experience with all co-players. Further generalizations are conceivable. For example, players may defect against all their co-players as long as at least one of their automata is in the D state, or they may remember more than their co-player’s last action^26,71^. Under crosstalk, the players’ individual games are no longer considered in isolation, but they are embedded into the context of all concurrently ongoing interactions. Crosstalk requires stronger mechanisms for forgiveness especially in a more highly connected world. A harsh retaliator such as Tit-for-Tat is particularly unable to deal with crosstalk. This is an interesting message for our current society.

## Methods

### Computer simulations

To simulate the effect of crosstalk on the cooperation dynamics among players with fixed strategies, we consider a population of size *N* playing a repeated Prisoner’s Dilemma (PD). The population is given by a graph where the nodes represent the players, and the edges reflect all possible interactions between players. Only players connected by an edge can be paired to play the PD. Players use separate two-state automata for each of their neighbors on the interaction graph^41^. The two states of each automaton are labeled C (cooperation) and D (defection). These states are updated according to the player’s reactive strategy (*p*, *q*), see Supplementary Figs. 1 and 2. The parameter *p* denotes the probability to move to state *C* if the co-player has cooperated in the previous game (whereas the complementary probability 1 − *p* gives the likelihood to move to state *D*). Similarly, *q* denotes the probability to move to state C if the co-player has defected (and 1 − *q* is the respective probability to move to state D).

In each round, an edge of the interaction graph is chosen uniformly at random. A single PD is played among the two players adjacent to the chosen edge. With probability 1 − *γ* a player acts according to the respective automaton state associated with this co-player; with probability *γ* crosstalk occurs and the player refers to the state of the automaton updated in her last interaction instead. After the game, the automata states are updated according to the game outcome and the players’ strategies. This elementary step is then iterated for a large number of rounds. For the simulation results depicted in Supplementary Fig. 5, we simulated 4000 games per realization (on average 500 games per player) and averaged across 10^4^ realizations to obtain the stationary payoff of GTFT and ALLD players for a given population structure and crosstalk rate.

So far, we assumed that every edge in the interaction graph is chosen with the same probability. However, some interactions can occur with a higher frequency than others. To investigate the effects of different interaction frequencies, we studied the spread of defective behavior in a population of GTFT players populating a 5 × 5 lattice (Supplementary Fig. 13). We increased the interaction probability of all players on the central horizontal line by 10-fold (see orange edges) and observe how defective behavior spreads much faster along the horizontal axis than along the vertical axis. Within the analytical framework, interaction probabilities *w*_*ij*_ are given by the connectivity matrix *W* (see next section for details).

In the second studied type of crosstalk, again with probability *γ* crosstalk occurs and the player refers to the state of a random automaton, chosen from all her interaction partners with equal probability (Supplementary Figs. 2 and 10).

### Analytical derivation of steady-state payoffs

To derive an explicit representation for the payoffs that players receive in the long run, we suppose that each player *i* adopts some fixed reactive strategy (*p*_*i*_, *q*_*i*_). The population structure is given by an *N* × *N* connectivity matrix *W* = (*w*_*ij*_). The entries *w*_*ij*_ give the probability that the next interaction in that population occurs between players *i* and *j*. In particular, the connectivity matrix *W* is symmetric *w*_*ij*_ = *w*_*ji*_, and satisfies *w*_*ii*_ = 0 and $\mathop {\sum}\nolimits_{i < j} w_{ij} = 1$. As in the computer simulations, we focus on networks in which each link is played with equal probability. That is, if player *i* and *j* are connected, then *w*_*ij*_ = *w* for some constant *w* > 0 that depends on the network structure, but is independent of the players *i* and *j*. For example, because there are *N*(*N* − 1) / 2 different links in a complete graph, well-mixed populations can be represented by a connectivity matrix with *w*_*ij*_ = 2 / (*N*(*N* − 1)) for all *i* ≠ *j*. As another example, populations on a cycle are represented by *w*_*ij*_ = 1/*N* if ${i}$ and *j* occupy neighboring sites, and *w*_*ij*_ = 0 for all other *i*, *j*.

Let $\bar w_i = \mathop {\sum}\nolimits_{j = 1}^N w_{ij}$ denote the probability that the next interaction in the population involves player *i*. Moreover, let $y_{ij}^t$ be the probability that player *i* is in state *C* against player *j* at time *t*, and let $y_i^t$ be the probability that player *i* is in state *C* with respect to her previous co-player. We can calculate $y_{i,j}^{t + 1}$ using the following recursion

1 $$\begin{array}{*{20}{l}} {y_{ij}^{t + 1}} \hfill &  = \hfill & {\underbrace {\left( {1 - w_{ij}} \right)y_{ij}^t}_{{\mathrm{If}}\,{\mathrm{player}}\,i\,{\mathrm{did}}\,{\mathrm{not}}\,{\mathrm{interact}}\,{\mathrm{with}}\,{\mathrm{player}}\,j\,{\mathrm{in}}\,{\mathrm{previous}}\,{\mathrm{round}}}} \hfill \\ {} \hfill & + \hfill & {\underbrace {(1 - \gamma )w_{ij} \cdot \left( {y_{ji}^tp_i + \left( {1 - y_{ji}^t} \right)q_i} \right)}_{{\mathrm{Players}}\,i\,{\mathrm{and}}\,j\,{\mathrm{interacted}},\,{\mathrm{and}}\,{\mathrm{player}}\,j{\prime}{\mathrm{s}}\,{\mathrm{action}}\,{\mathrm{was}}\,{\mathrm{not}}\,{\mathrm{subject}}\,{\mathrm{to}}\,{\mathrm{crosstalk}}}} \hfill \\ {} \hfill & + \hfill & {\underbrace {\gamma w_{ij} \cdot \left( {y_j^tp_i + \left( {1 - y_j^t} \right)q_i} \right)}_{{\mathrm{Players}}\,i\,{\mathrm{and}}\,j\,{\mathrm{interacted}},\,{\mathrm{and}}\,{\mathrm{player}}\,j{\prime}{\mathrm{s}}\,{\mathrm{action}}\,{\mathrm{was}}\,{\mathrm{subject}}\,{\mathrm{to}}\,{\mathrm{crosstalk}}}.} \hfill \end{array}$$

To calculate the player’s long run cooperation frequencies, we note that in the steady state, cooperation rates are independent of the time period, and hence $y_{ij}^{t + 1} = y_{ij}^t = :y_{ij}$. Moreover, because interactions are fully random, the stationary probabilit*y y*_*i*_ that player *i* is in state C with respect to her previous co-player simplifies to $y_i = \mathop {\sum}\nolimits_{k = 1}^N \frac{{w_{ik}}}{{\bar w_i}}y_{ik}$, which is the weighted average that player *i* is in state C with respect to a random co-player. In that case, we can rewrite Eq. (1) as

2 $$y_{ij} - (1 - \gamma )\left( {p_i - q_i} \right)y_{ji} - \gamma \left( {p_i - q_i} \right)y_j = q_i.$$

The Eqs. (2) represent a system of *N*(*N* − 1) linear equations in the unknowns *y*_*ij*_ with *i* ≠ *j*. By solving this inhomogeneous system, we can calculate the stationary frequency $\hat y_{ij}$ to find player *i* in state *C* with respect to player *j*. Given the stationary frequencies $\hat y_{ij}$, we can calculate the payoff *π*_*i*_ of player *i* by averaging over all co-players,

3 $$\pi _i = \mathop {\sum}\limits_{j = 1}^N \frac{{w_{ij}}}{{\bar w_i}} \cdot \left[ {\left( {(1 - \gamma )\hat y_{ji} + \gamma \hat y_j} \right) \cdot b - \hat y_{ij} \cdot c} \right].$$

We note that this method applies to general crosstalk rates *γ*, general population structures, and general population compositions (e.g., populations with more than two different strategies present). As shown in Supplementary Fig. 5, these analytically derived payoffs are in excellent agreement with the computer simulations. In the Supplementary Note 1, we show how Eqs. (2) and (3) can be further simplified for well-mixed populations. In that case, we can also provide explicit expressions for how generous cooperative strategies of the form (1, *q*) are allowed to be to resist invasion of ALLD (as depicted in Fig. 3c, d).

### Setup of the evolutionary simulations

To explore the evolution of strategies under crosstalk, we consider a simple model of cultural evolution, the pairwise comparison process^50,51^. As common in studies on the evolution of strategies in repeated games^10–25,40,41^, we assume a separation of time scales: the time it takes individuals to play their repeated games is short compared to the evolutionary timescale at which individuals adopt new strategies. This assumption allows us to use the players’ stationary payoffs, as given by Eq. (3), when simulating the evolutionary trajectory of a population.

For the evolutionary simulations, we consider a population with fixed population structure and fixed crosstalk rate *γ*. In each evolutionary time step, there are two possible events, imitation or random strategy exploration. To model imitation events, we assume that two individuals are randomly drawn from the population. We refer to these two individuals as the “learner” and the “role model”, respectively. Herein, we aim to compare the effect of crosstalk across different population structures. To allow for a fair comparison, we assume that, while payoffs are calculated for the given population structure, strategy updating occurs globally. As a consequence, the learner and the role model do not need to be neighbors in the direct interaction network. With this assumption, we rule out the formation of cooperative clusters, which would additionally favor the evolution of cooperation in networks with a low degree^44^. After selecting the learner and the role model, their payoffs *π*_L_ and *π*_R_ are calculated according to Eq. (3). We assume that the learner adopts the role model’s strategy with probability *ρ* = [1 + exp(−*s*(*π*_R_ − *π*_L_))]^−1^. The parameter *s* ≥ 0 measures the strength of selection. When selection is weak, $s \ll 1$, payoffs are largely irrelevant for imitation and the imitation probability approaches 1/2, irrespective of the players’ strategies. When selection is strong, $s \gg 1$, players tend to adopt only those strategies that yield a higher payoff than their own strategy. In addition to these imitation events, we allow for random strategy exploration. When such an exploration event occurs, one player is randomly drawn from the population. This player then adopts a new strategy (*p*, *q*), which is uniformly drawn from all reactive strategies. Following the approach of Imhof and Nowak^23^, we assume that these exploration events are rare. As a consequence, the population is homogeneous most of the time. Only occasionally, a mutant strategy enters the population due to random strategy exploration. This mutant strategy than either goes extinct or fixes before the next exploration event occurs. By simulating this process over a long timespan, we can record how often the population applies certain strategies (*p*, *q*), and we can compute the resulting average cooperation rate over an evolutionary timescale. In Fig. 4, we show corresponding results for the cycle, the square lattice, and for the complete graph, assuming parameter values of population size *N* = 16, benefit *b* = 10, cost *c* = 1, and selection strength *s* = 1. Other parameter values lead to qualitatively similar results, provided that selection is sufficiently strong and that the benefit of cooperation is sufficiently high to allow for the evolution of cooperation. In the Supplementary Note 1, we show that analogous results apply when we consider a birth-death process instead of the pairwise imitation process considered herein.

### Data availability

No data sets were generated during this study.

## Electronic supplementary material

Supplementary Information
