# Supplementary material for: Crosstalk in concurrent repeated games impedes direct reciprocity and requires stronger levels of forgiveness
Source: Nat Commun. 2018 Feb 7;9:555. doi: 10.1038/s41467-017-02721-8 (PMC5803203; doi:10.1038/s41467-017-02721-8)
Supplement: Supplementary file 1 — Supplementary Information [file 41467_2017_2721_MOESM1_ESM.pdf]

Supplementary Figures

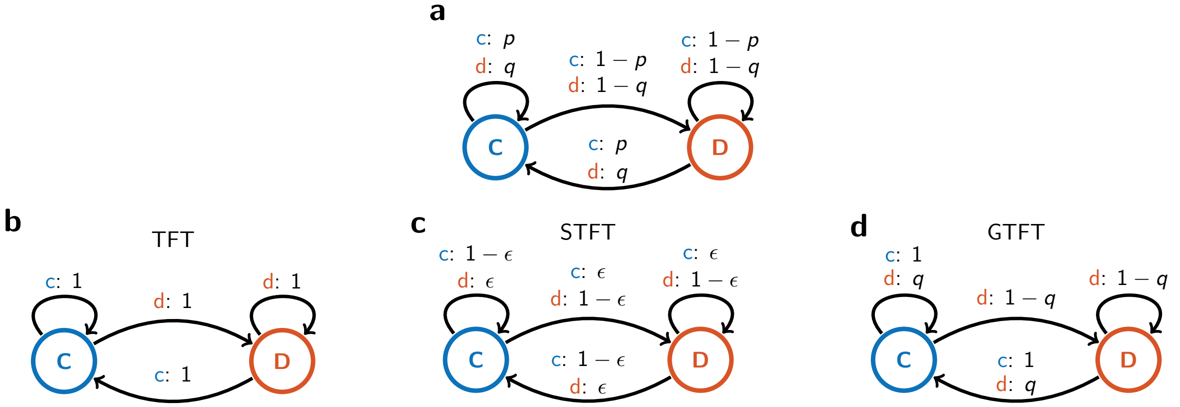

Supplementary Figure 1: Reactive strategies implemented by two-state automata. The blue circle depicts the state where the player cooperates (c) and the red circle depicts the state where the player defects (d) in the next game. After a game, depending on the action (c or d) of the co-player, the state changes according to the given probabilities. **a** | A stochastic reactive strategy is encoded by the tuple (p, q) denoting the probability to cooperate if the co-player in the previous round either cooperated or defected, respectively. **b**-**d** | The well-known strategies Tit-for-Tat (TFT), Stochastic Tit-for-Tat (STFT), and Generous Tit-for-Tat (GTFT; $0 < q < 1$) implemented by stochastic two-state automata.

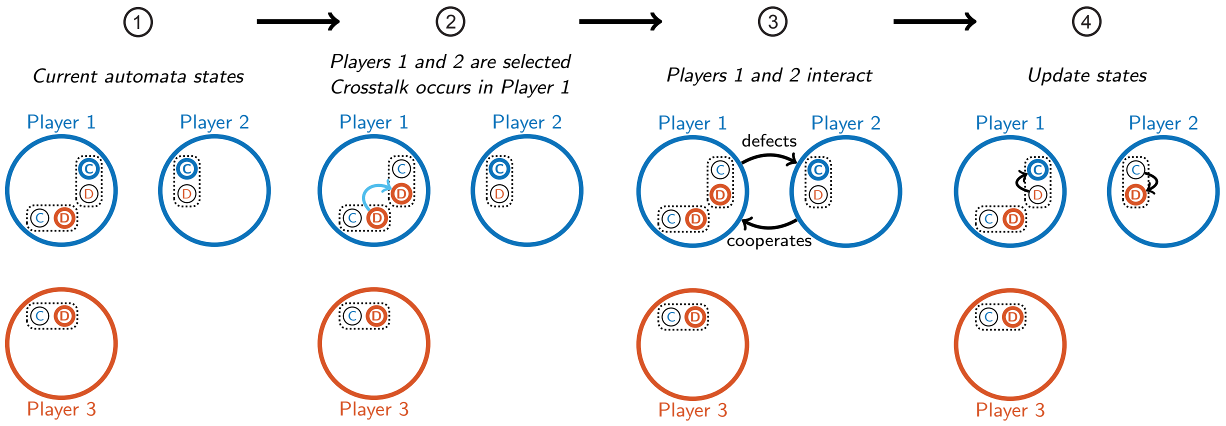

Supplementary Figure 2: Crosstalk in concurrent repeated games. Players (large circles) use reactive strategies implemented by a separate two-state automata for each interaction partner. The current state is emphasized in bold font (panel 1). Two random players (here players 1 and 2) are selected to play a PD (Prisoner's Dilemma; panel 2). Crosstalk between independent automata within the involved players 1 and 2 happens with a small probability γ. Crosstalk might change the action in the following interaction. By chance, state D of the automaton implementing the interaction of player 1 with player 3 is copied to the automaton implementing the interaction of player 1 with player 2 (indicated by a blue arrow). Here player 1 defects (due to crosstalk) and player 2 cooperates (panel 3). The states of the automata are updated according to the player's strategy (panel 4). Player 1 plays TFT (Tit-for-Tat) and moves to state C. Player 2 plays TFT and moves to state D.

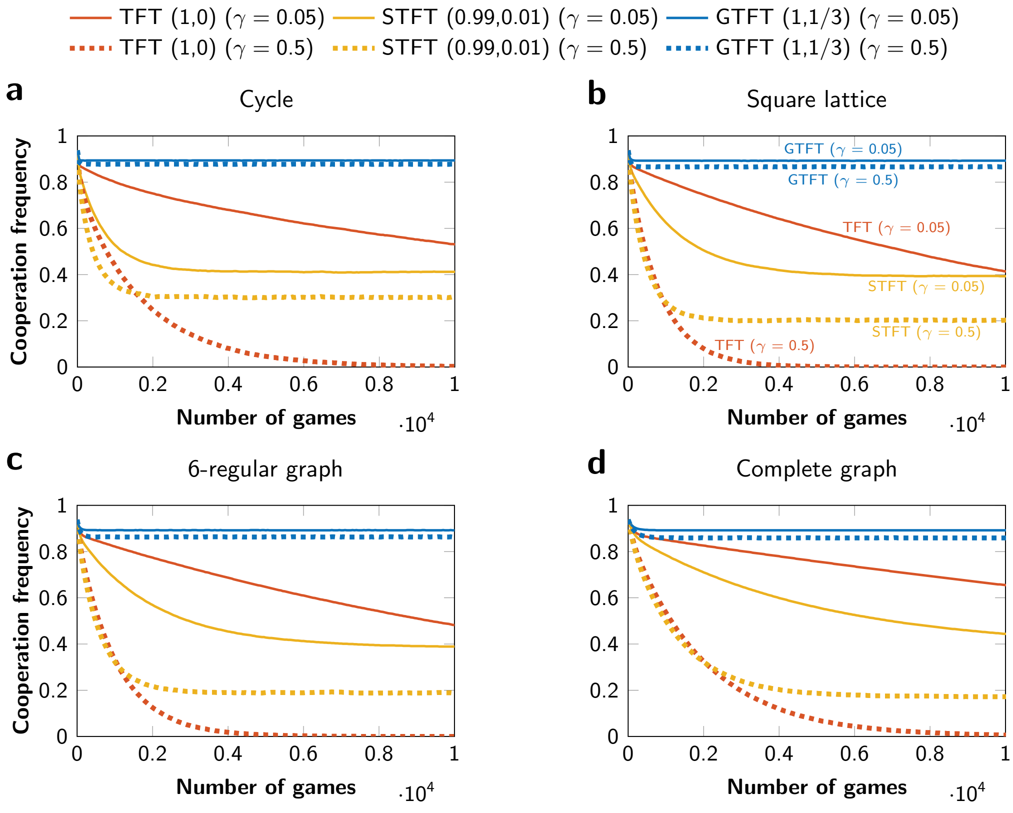

Supplementary Figure 3: Generous Tit-for-Tat (GTFT) maintains high levels of cooperation in the presence of crosstalk over time. The level of cooperation is determined by the frequency that players are in the cooperative state, averaged over all players in the population. Full lines correspond to a crosstalk rate of $\gamma = 0.05$ and dotted lines correspond to a crosstalk rate of $\gamma = 0.5$. **a-d** | Since TFT (Tit-for-Tat) is not an error-correcting strategy, its cooperation frequency converges to zero for any $\gamma > 0$. Stochastic Tit-for-Tat (STFT) can secure a basic level of cooperation as it sometimes forgives defection. Across all population structures GTFT (Generous Tit-for-Tat) maintains a high level of cooperation. High crosstalk rates lead to a faster spreading of defective behavior for both TFT and STFT whereas population structures with a low connectivity delay the spreading of defection (e.g., cycle or square lattice). All players use a given conditional cooperative strategy except one random player always defects (ALLD). Number of players is $N = 16$ (one of those is the ALLD player). Simulation results are averages over 10^4 realizations.

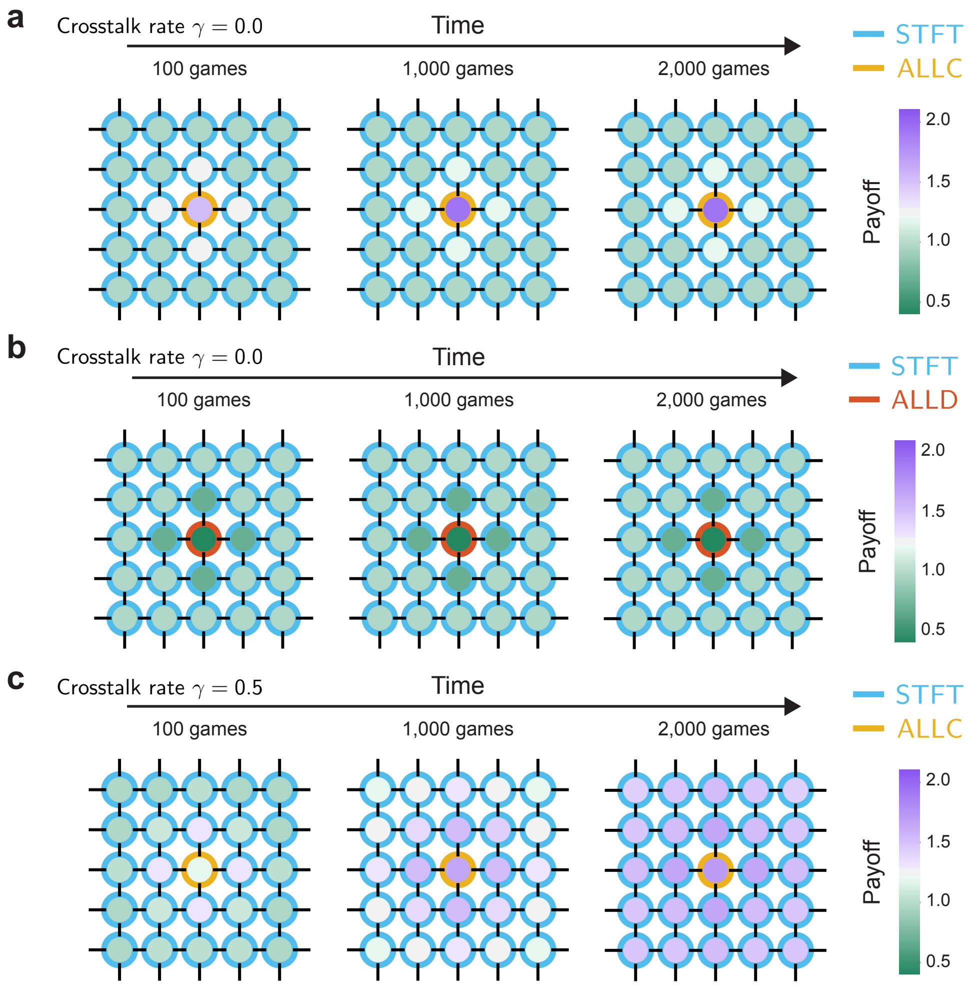

Supplementary Figure 4: Cooperative and defective behavior can spread with crosstalk but not without. Twenty-four erroneous conditional cooperators (STFT $p = 0.999$, $q = 0.001$; blue framed nodes) and one ALLD (red framed node, placed in the center) or ALLC (yellow framed node) player populate a 5x5 lattice. The fill color of the nodes depicts the expected payoff of the players after 100, 1,000 and 2,000 games. **a-b** | In the absence of crosstalk ($\gamma = 0.0$) cooperative and defective behavior can not spread. The erroneous ALLC ($p = 0.999$, $q = 0.999$) or ALLD ($p = 0.001$, $q = 0.001$) player only affect the payoff of its STFT neighbors. **c** | In the presence of crosstalk ($\gamma = 0.5$) cooperation spreads from the ALLC player via crosstalk to all STFT players. We assume that in the first round, the STFT players are equally likely to cooperate or to defect, which is their stationary cooperation frequency in a homogeneous population of STFT players. Parameter values: benefit $b = 3$, and cost $c = 1$.

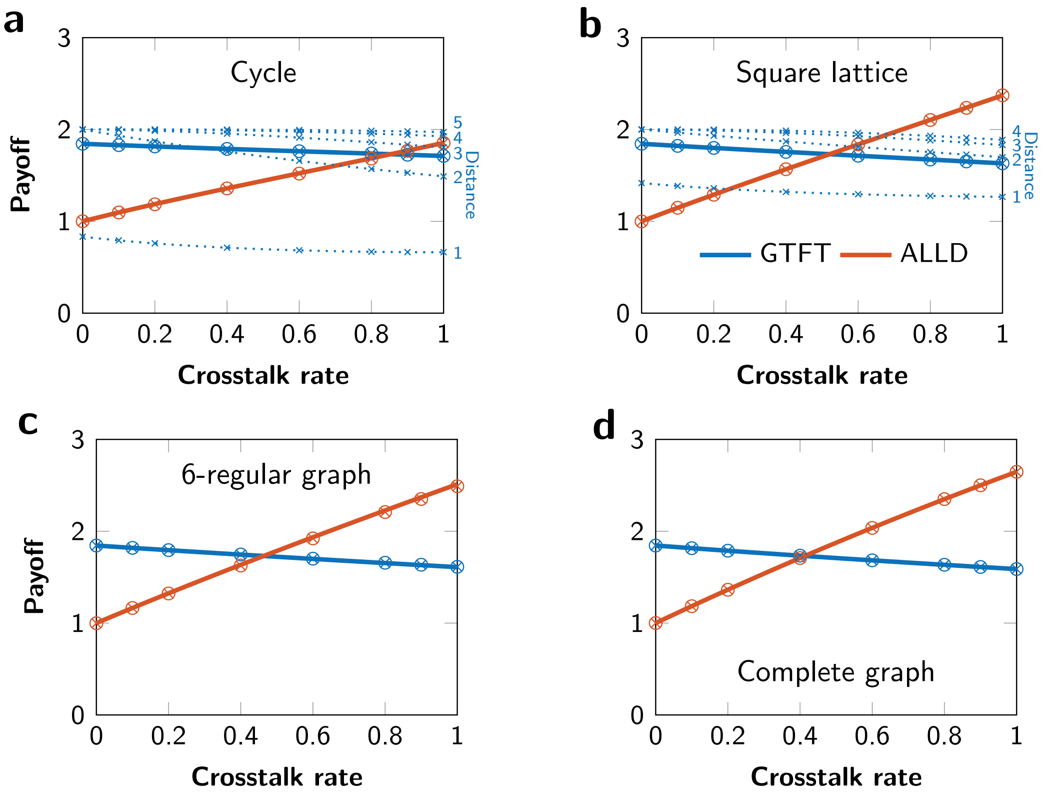

Supplementary Figure 5: Stationary payoff varies across population structures and crosstalk rates. Stationary payoff of GTFT ($p = 1, q = 1/3$) and ALLD $(0, 0)$ players versus the crosstalk rate in different population structures. One ALLD player is randomly placed on the graph, among $N - 1$ GTFT players. Full lines show numerically exact results for the average payoff of all GTFT players (blue) and of the ALLD player (red) in the steady state. Dotted lines show the steady state payoff of individual players with a given distance to the ALLD player. Circles and crosses show the respective simulation results. The larger the distance of a GTFT player to an ALLD player, the less likely a player's payoff is affected by the ALLD player. Players with distance 1 are adjacent to the ALLD player. **a–d** | On the cycle, the average payoff of the GTFT players exceeds the defector's payoff up to a crosstalk rate of $\gamma \approx 0.85$, whereas for well-mixed populations the critical crosstalk rate is much lower, $\gamma \approx 0.41$. The other two population structures exhibit crosstalk thresholds in between these two extremes. Parameter values: number of players $N = 16$ (one ALLD player), benefit $b = 3$, and costs $c = 1$. Simulation results are averages over 10^4 realizations.

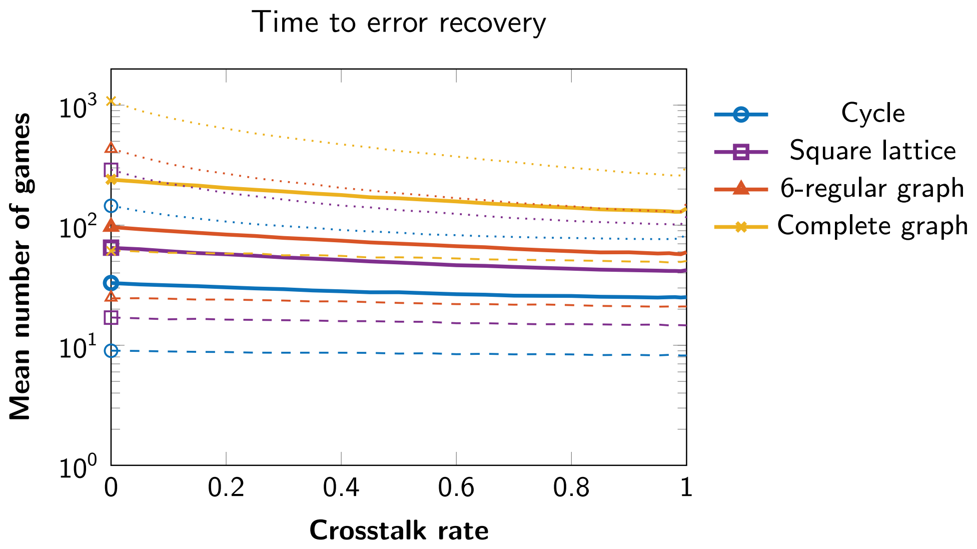

Supplementary Figure 6: Mean time until a population of conditional cooperators returns to full cooperation after a single error. Higher crosstalk rates (γ) as well as probabilities to cooperate after defection (q) decrease the number of games to recover from an error such that the whole population returns to full cooperation. For the case of $\gamma = 0$, analytical results are denoted by blue circles for the cycle, purple squares for the square lattice, red triangles for the 6-regular graph, and yellow crosses for the complete graph (see Section 2.1 for further details). Parameter values: number of GTFT players $N = 16$, all GTFT players ($p = 1$; full lines: $q = 1/3$, dotted lines: $q = 0.1$; dashed lines: $q = 2/3$), benefit $b = 3$, and costs $c = 1$. Simulation results are averages over 10^5 realizations.

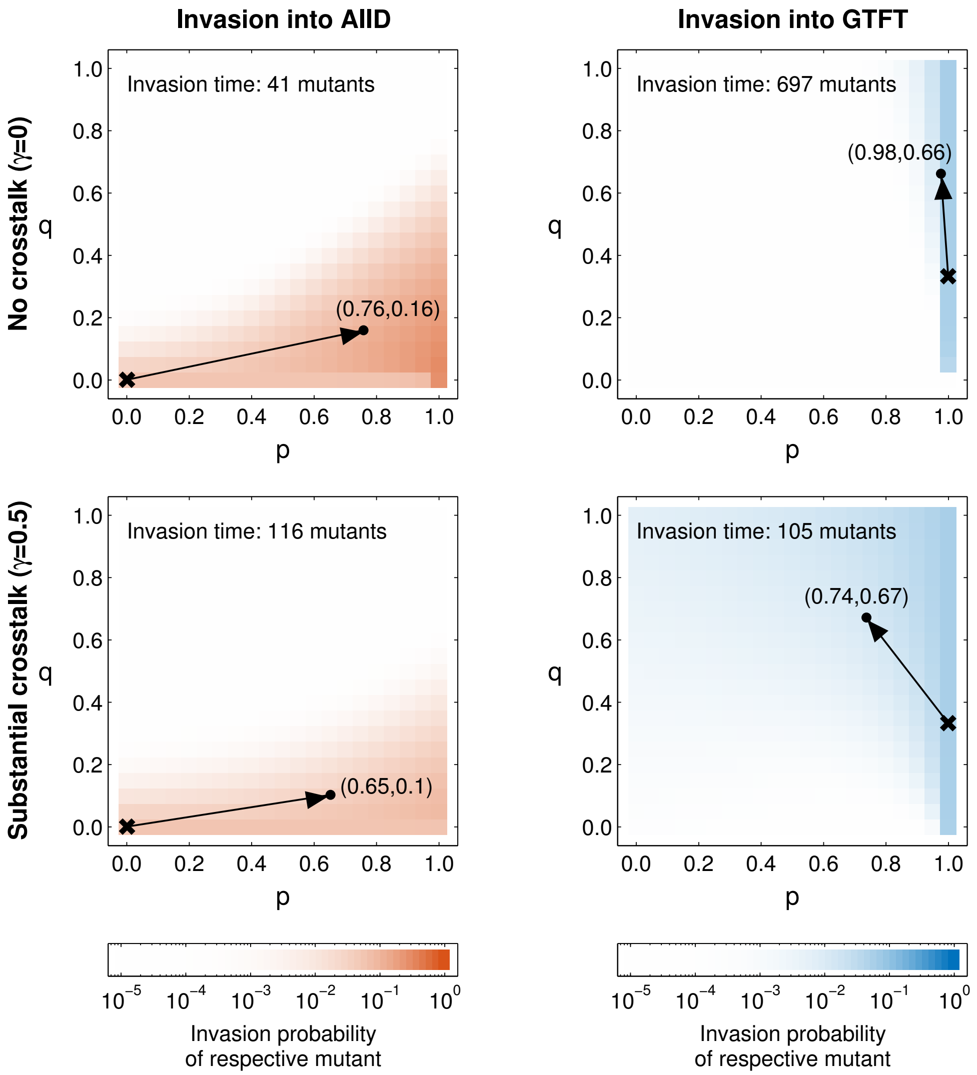

Supplementary Figure 7: Higher crosstalk rates simplify the invasion of selfish strategies into GTFT. For two different resident strategies, ALLD and GTFT, we have calculated how easily mutants can invade. To this end, we have considered a fine grid of mutant strategies (p, q) with $p, q \in \{0, 0.005, 0.010, \ldots, 1\}$. For each of these mutant strategies, we have calculated its fixation probability into the respective resident strategy. The value of the fixation probability is represented by the color of the respective square at (p, q). In addition, we have simulated how many mutant invasions the resident strategies can resist if mutants are randomly drawn from that grid (reported in the upper left of each panel). We have also recorded the average trait values of successful mutants (indicated by the arrow). We find that ALLD is typically invaded by conditionally cooperative strategies, and that the invasion time increases with the crosstalk rate. For GTFT, we find that in the absence of crosstalk, it takes a considerable number of mutants until the first mutant reaches fixation. Moreover, the successful mutant is typically a cooperative strategy itself. As the crosstalk rate increases, however, the invasion time into GTFT decreases, and successful mutants do no longer need to be cooperative. Parameters: population size $N = 16$, benefit $b = 10$, cost, $c = 1$, selection strength $s = 1$. The strategies are subject to small amounts of noise, ALLD $= (0.001, 0.001)$ and GTFT $= (0.999, 0.333)$.

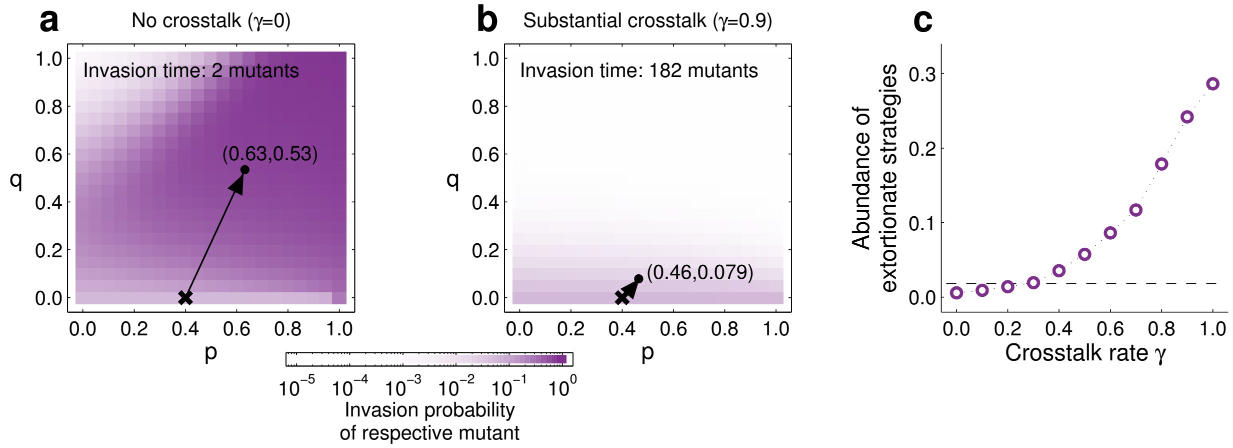

Supplementary Figure 8: Higher crosstalk rates can stabilize extortion. a,b | Similar to the invasion analysis for ALLD and GTFT reported in **Supplementary Fig. 7**, we explored how many mutant invasions it takes until an extortionate resident population is successfully replaced. Without crosstalk, extortionate strategies are quickly replaced by more cooperative strategies. This result is in line with previous observations that extortion is unstable in typical models of direct reciprocity [1]. However, once there is substantial crosstalk, it takes more mutant strategies until an extortionate resident population is invaded, and successful mutants are similar to the extortionate strategy. **c** | To quantify the overall success of extortion in well-mixed populations, we have recorded how often the evolutionary process visits a δ-neighborhood of the set of all extortionate strategies (see also Refs. [1, 2]). For comparison, the dashed line indicates how often this neighborhood is visited in the case of neutral evolution (when the selection strength s is zero). As the crosstalk rate approaches $\gamma = 1$, the set of extortionate strategies is visited more than 10 times more often than expected under neutrality. Thus, when crosstalk is common, selection favors extortionate strategies. Parameters: population size $N = 16$, $b = 10$, $c = 1$, $s = 1$. For the invasion analysis, we have used the extortionate resident strategy (0.4,0), and for the δ-neighborhood, we have used $\delta = 0.02$.

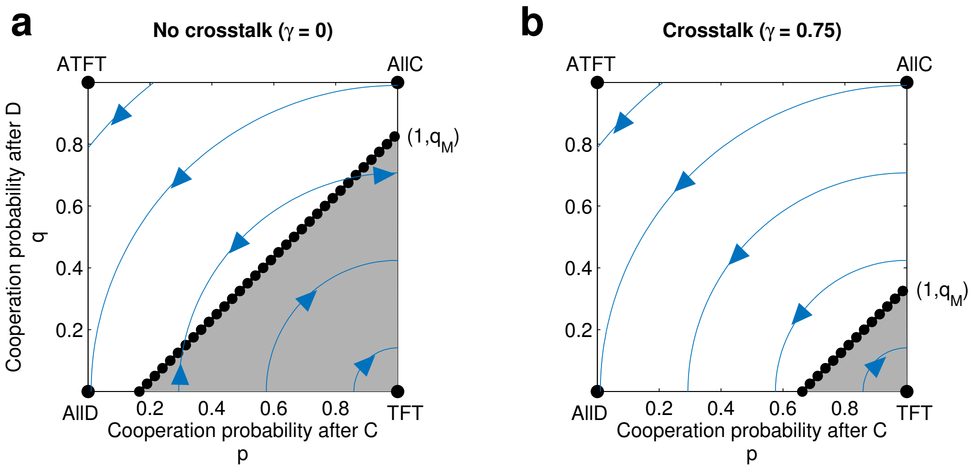

Supplementary Figure 9: Deterministic adaptive dynamics of crosstalk. Blue curves represent numerically computed solutions of the adaptive dynamics, Eq (S13), for two different crosstalk scenarios. The state space consists of all pairs (p, q) in the unit square. The four corners of the square correspond to ALLD (0,0), Tit-For-Tat (1,0), ALLC (1,1) and Anti-Tit-for-Tat (0,1). The black-dotted line is the set of singular points, as given by Eq. (S11); the grey area below that line is the cooperation-rewarding zone. As the crosstalk rate γ increases from 0 to 0.75, this cooperation-rewarding zone shrinks considerably; most initial population configurations lead to a state in which everyone defects. Parameter values: population size $N = 16$, benefit $b = 10$, cost $c = 1$.

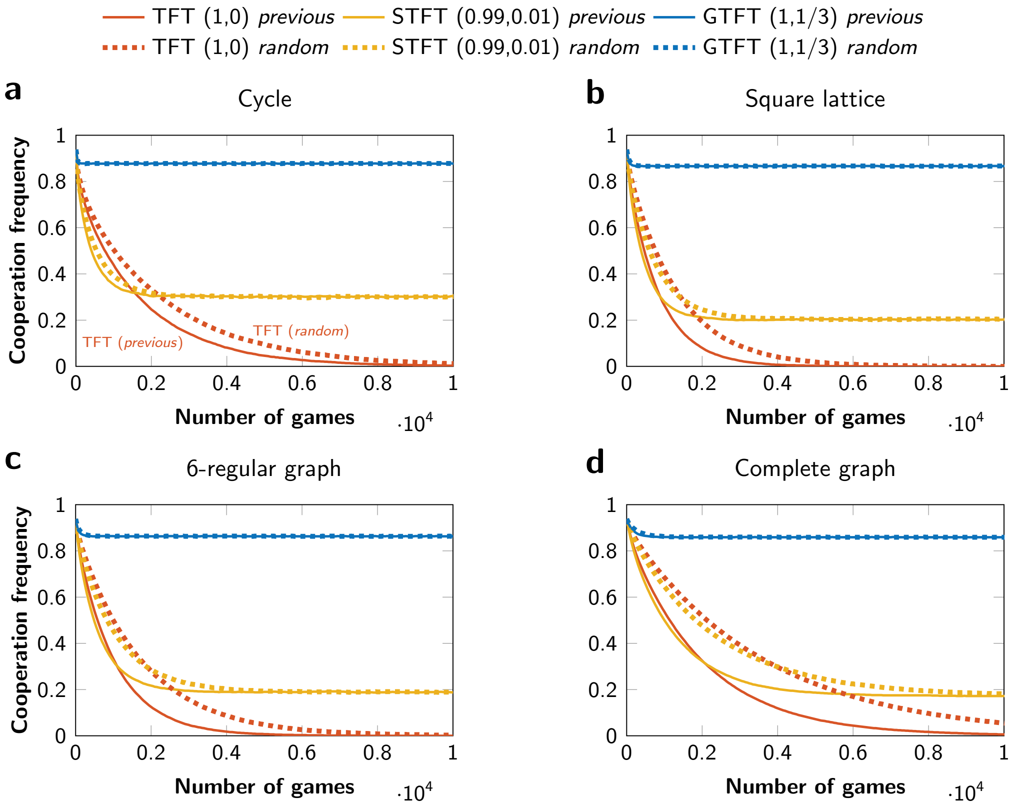

Supplementary Figure 10: Dynamics of cooperation for two different types of crosstalk. For the original model used in the main text, we have assumed that crosstalk leads a player to refer to the automaton of the $previous$ interaction. Here, we compare the cooperation dynamics of this original model (see also Fig. 3) to an alternative implementation of crosstalk, according to which players refer to a $random$ automaton. While the behavioral dynamics can be different under the two types of crosstalk, the stationary distributions are identical. Higher crosstalk rates, densely connected populations and previous interaction crosstalk (full lines) accelerate spread of defective behavior. The number of players is $N = 16$ (one of those is the ALLD player), and the crosstalk rate is $\gamma = 0.5$. Simulation results are averages over 10^4 realizations.

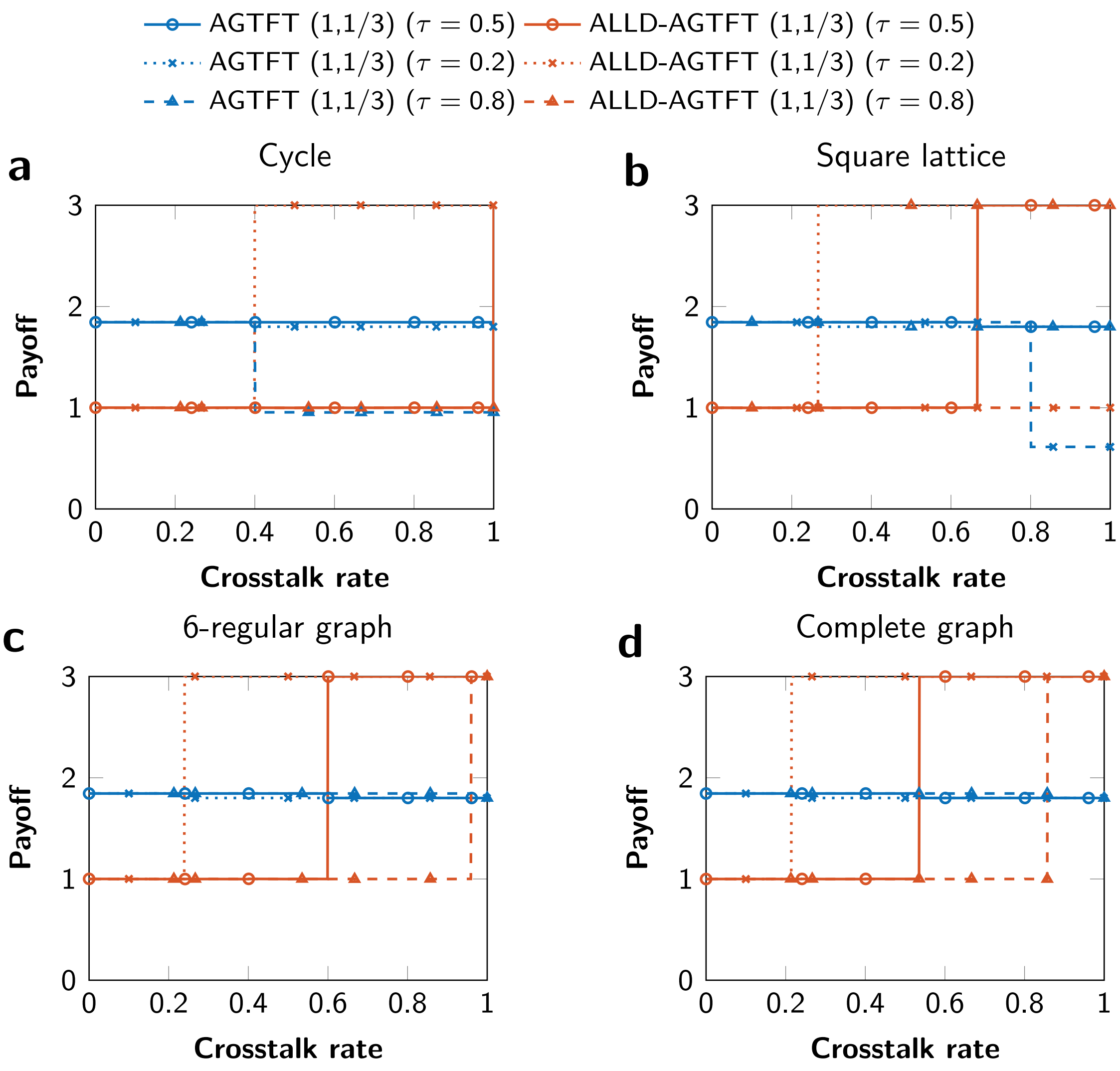

Supplementary Figure 11: Stationary payoff of Aggregate Generous Tit-for-Tat (AGTFT) and ALLD players over the crosstalk rate across different population structures. Generalizing the strategy space of reactive strategies to aggregate reactive strategies allowed us to condition the next action on the aggregate received cooperation in the previous round (see **Sec. 2.2** for details). Simulation results accurately match our analytical results (see **Supplementary Fig. 12** and **Sec. 2.2**). One ALLD player is randomly placed on the graph, among $N-1$ GTFT players. Parameter values: number of players $N=16$, all AGTFT players ($p=1$, $q=1/3$; full lines: $\tau=0.5$, dotted lines: $\tau=0.2$; dashed lines: $\tau=0.8$), benefit $b=3$, and costs $c=1$. Simulation results are averages over 10^4 realizations.

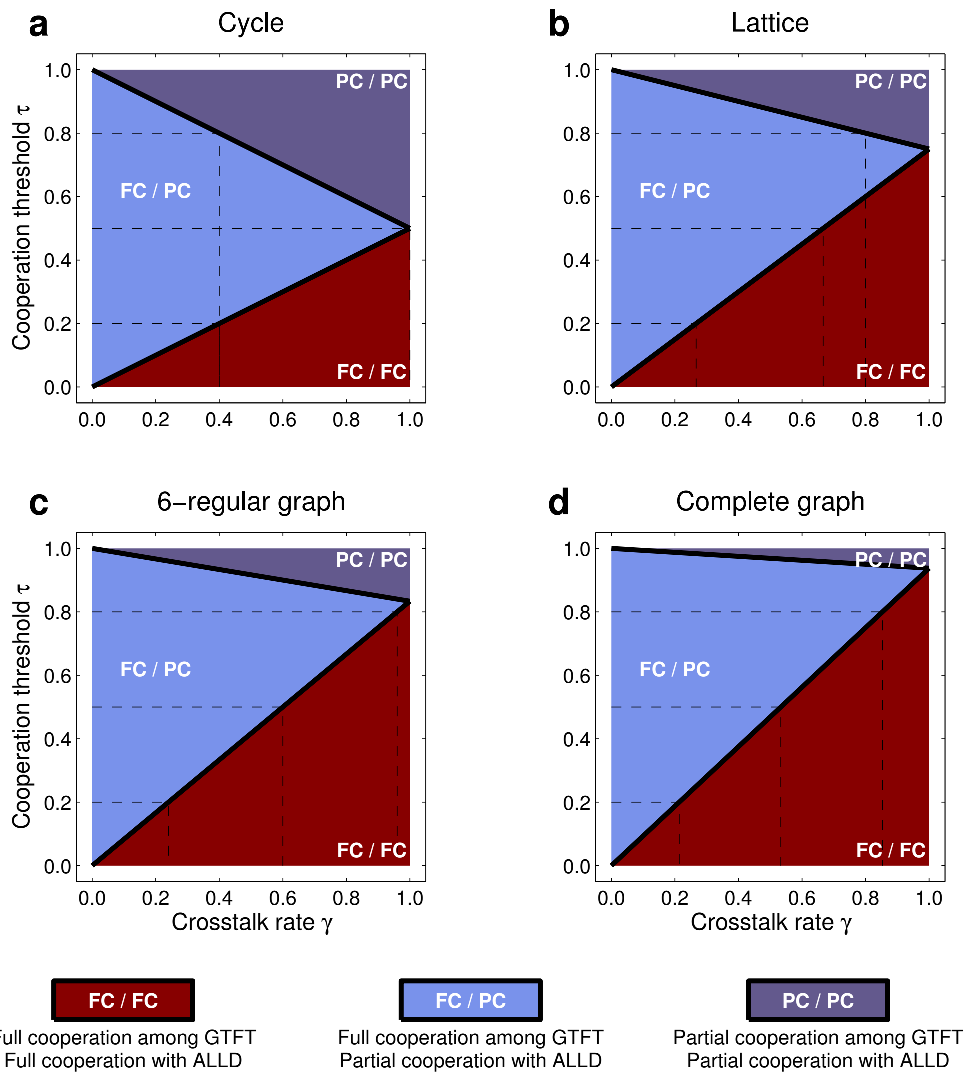

Supplementary Figure 12: Aggregate Generous Tit-for-Tat (AGTFT) requires intermediate cooperation thresholds τ to be most robust against invasion of defectors. We consider four different population structures, and as in **Supplementary Fig. 11**, we assume there is one ALLD players and $N-1$ players with strategy AGTFT. Depending on the region in the (γ, τ) parameter space, there are three different qualitative outcomes. *(i) FC/FC:* In this case, the AGTFT players cooperate with everyone. As a consequence, the defector gets a higher payoff than all residents. *(ii) FC/PC:* The AGTFT players fully cooperate among each other, but they only partially cooperate with the ALLD player. In this case AGTFT is stable against ALLD if the conditional cooperation probability q is sufficiently low. *(iii) PC/PC:* Here, the AGTFT do no longer fully cooperate among themselves. The analytically derived boundaries of the three parameter regions match the numerically found results in **Supplementary Fig. 11**. Parameter values: number of players $N = 16$.

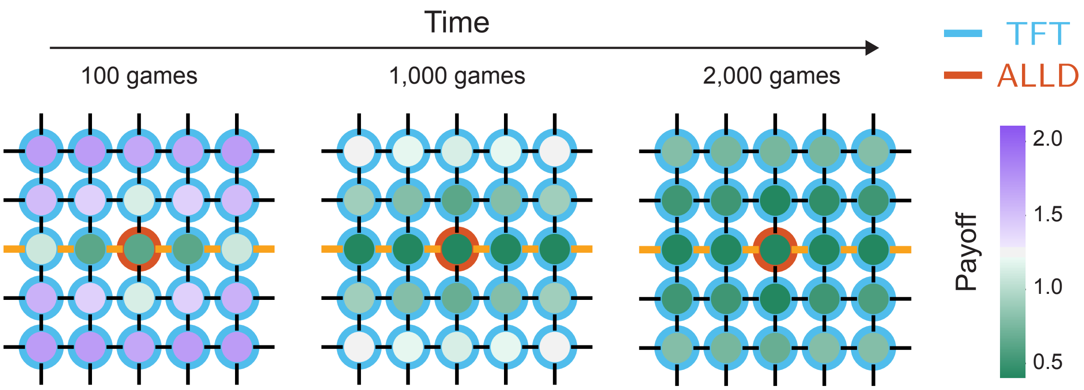

Supplementary Figure 13: Increased interaction frequencies boost effect of crosstalk. Twenty-four conditional cooperators (blue framed nodes, panels) and one ALLD (Always-Defect) player (red framed node, placed in the center) populate a 5x5 lattice. Players connected by an orange line have a 10-fold increased interaction probability. Defection spreads faster due to the increased interaction probability along the central, horizontal line of players. The fill color of the nodes depicts the expected payoff of the players after 100, 1,000 and 2,000 games. Parameter values: crosstalk rate $\gamma = 0.5$, benefit $b = 3$, and cost $c = 1$. For GTFT (defined by $p = 1$ and $0 < q < 1$), we used $q = 1/3$.

Supplementary Note 1

In Section 1, we provide further analytical results for our model of crosstalk in the special case of well-mixed populations. Specifically, we describe a more efficient algorithm to calculate steady-state payoffs when only two different strategies are present. Using this algorithm, we can calculate explicitly which strategies (p, q) are able to resist invasion by any other mutant strategy (p', q'). Moreover, the algorithm allows us to explore the adaptive dynamics of the system for any crosstalk rate.

In Section 2, we present additional results that hold for any population structure. We compute the time that a cooperative population needs to recover from an isolated defection event. Moreover, we introduce a model of crosstalk that allows players to react to the aggregate cooperation received across all their co-players. Finally, we argue that the evolutionary results presented in the main text remain unchanged if we consider a birth-death process instead of an imitation process.

1 Analytical results for well-mixed populations

1.1 Efficient calculation of payoffs in the special case of two strategies

In the main text, we have derived the following linear system to capture the players' cooperation frequencies in the steady state,

$$y_{ij} - (1-\gamma)(p_i - q_i)y_{ji} - \gamma(p_i - q_i)y_j = q_i. \tag{S1}$$

In this equation, the unknowns y_{ij} represent the probability that player i cooperates against player j in the steady state, and (p_i, q_i) is the reactive strategy of player i. By solving this linear system, we can calculate payoffs in small and intermediate-sized populations. However, as populations become large, the computational effort increases quadratically in the population size N. We thus derive a more efficient algorithm for the complete graph, assuming that there are only two different strategies present in the population. Suppose that k individuals use strategy (p_1, q_1), whereas the remaining $N-k$ individuals use strategy (p_2, q_2). Because the population structure is fully symmetric, we can assume that players with the same strategy receive the same payoff. Hence, we set $y_{ij} = y_{i'j'}$ in Eq. (S1) whenever the strategies of player i and i' and the strategies of player j and j' coincide. This implies a drastic simplification: instead of having to consider all $N \cdot (N-1)$ possible combinations of players, we only need to consider all 4 possible combinations of strategies present in the population. That is, the linear system (S1) simplifies to the linear system $M\mathbf{y} = \mathbf{x}$,

where M is the 4×4 matrix

$$M = \begin{pmatrix} 1-\left(1-\frac{N-k}{N-1}\gamma\right)r_1 & -\frac{N-k}{N-1}\gamma r_1 & 0 & 0 \\ 0 & 1 & -\left(1-\frac{N-k-1}{N-1}\gamma\right)r_1 & -\frac{N-k-1}{N-1}\gamma r_1 \\ -\frac{k-1}{N-1}\gamma r_2 & -\left(1-\frac{k-1}{N-1}\gamma\right)r_2 & 1 & 0 \\ 0 & 0 & -\frac{k}{N-1}\gamma r_2 & 1-\left(1-\frac{k}{N-1}\gamma\right)r_2 \end{pmatrix}, \quad \text{(S2)}$$

with $r_i := p_i - q_i$, and $\mathbf{x} = (q_1, q_1, q_2, q_2)^T$. The solution vector $\hat{\mathbf{y}} = (\hat{y}_{11}, \hat{y}_{12}, \hat{y}_{21}, \hat{y}_{22})^T$ contains the respective steady-state frequencies $\hat{y}_{ij}$ for a player with strategy i to be in state C with respect to a co-player with strategy j. Using this vector, we can again calculate the expected payoffs of the two strategies as

$$\begin{aligned}
\pi_1 = &\left((1-\gamma)\left(\tfrac{k-1}{N-1}\hat{y}_{11}+\tfrac{N-k}{N-1}\hat{y}_{21}\right)+\gamma\left(\tfrac{k-1}{N-1}\tfrac{k-1}{N-1}\hat{y}_{11}+\tfrac{k-1}{N-1}\tfrac{N-k}{N-1}\hat{y}_{12}+\tfrac{N-k}{N-1}\tfrac{k}{N-1}\hat{y}_{21}+\tfrac{N-k}{N-1}\tfrac{N-k-1}{N-1}\hat{y}_{22}\right)\right)\cdot b \\
&-\left(\tfrac{k-1}{N-1}\hat{y}_{11}+\tfrac{N-k}{N-1}\hat{y}_{12}\right)\cdot c, \\
\pi_2 = &\left((1-\gamma)\left(\tfrac{N-k-1}{N-1}\hat{y}_{22}+\tfrac{k}{N-1}\hat{y}_{12}\right)+\gamma\left(\tfrac{k}{N-1}\tfrac{k-1}{N-1}\hat{y}_{11}+\tfrac{k}{N-1}\tfrac{N-k}{N-1}\hat{y}_{12}+\tfrac{N-k-1}{N-1}\tfrac{k}{N-1}\hat{y}_{21}+\tfrac{N-k-1}{N-1}\tfrac{N-k-1}{N-1}\hat{y}_{22}\right)\right)\cdot b \\
&-\left(\tfrac{N-k-1}{N-1}\hat{y}_{22}+\tfrac{k}{N-1}\hat{y}_{21}\right)\cdot c\,.
\end{aligned} \quad \text{(S3)}$$

We note that the computation time for the payoffs is now independent of the population size.

1.2 The optimal level of generosity

Based on the above method to calculate payoffs in well-mixed populations with two strategies, we can also analytically derive the most generous strategy $(1, q_M)$ and the most robust strategy $(1, q_R)$, as defined in the main text. To this end, we consider a population in which $N-1$ individuals adopt a cooperative strategy, whereas the remaining individual plays ALLD. If we set $(p_1, q_1) := (0, 0)$, $(p_2, q_2) := (1, q)$, and $k=1$, we can use Eq. (S3) to calculate the payoff of the single ALLD player as

$$\pi_D = bq\cdot(N-1)\frac{q(1-\gamma)+\gamma}{q(N-1-\gamma)+\gamma}\,. \quad \text{(S4)}$$

In contrast, the payoff of each cooperative player becomes

$$\pi_C = b - c - \big(b - c(1-q)\big)\frac{q(1-\gamma)+\gamma}{q(N-1-\gamma)+\gamma}\,. \quad \text{(S5)}$$

To calculate the most generous strategy that can resist invasion by ALLD we solve $\pi_D = \pi_C$, yielding

$$q_M = 1 - \frac{b+c(N-1)}{c+b(N-1)}\cdot\frac{1}{1-\gamma}\,. \quad \text{(S6)}$$

In particular, for no crosstalk and large populations ($\gamma = 0$ and $N \to \infty$), we recover the well-known probability $q_M = 1 - c/b$ [3, 4]. Because

$$\frac{\partial q_M}{\partial \gamma} = -\frac{b + c(N-1)}{c + b(N-1)} \cdot \frac{1}{(1-\gamma)^2} < 0, \tag{S7}$$

the maximum level of generosity is monotonically decreasing in γ. Thus, the more crosstalk, the less generous cooperative players need to be to still prevent the invasion of ALLD. The value of q_M becomes zero when

$$\gamma^* = \frac{(b-c)(N-2)}{c + b(N-1)} \leq 1 - c/b < 1 \ . \tag{S8}$$

In particular, only if the crosstalk rate satisfies $\gamma < \gamma^*$, we can hope for full cooperation to evolve in the complete graph.

Similarly, we can also calculate the most robust cooperative strategy, defined as the strategy $(1, q)$ that has the highest relative payoff advantage compared to a single ALLD mutant. By setting $\frac{\partial}{\partial q}(\pi_C - \pi_D) = 0$, we yield

$$q_R = -\frac{\gamma}{N-1-\gamma} + \sqrt{\frac{(N-2)\gamma\big((b-c)(N-1) - bN\gamma\big)}{\big(c + b(N-1)\big)(1-\gamma)(N-1-\gamma)^2}} \ . \tag{S9}$$

We note that q_R is zero for $\gamma = 0$ and for $\gamma = \gamma^*$, with γ^* as defined by Eq. (S8). In between, for $0 < \gamma < \gamma^*$, the value of q_R is positive. This means that as the crosstalk goes to zero, $\gamma \to 0$, we get $q_R \to 0$ and the strategy most robust against invasion by ALLD approaches TFT = $(1, 0)$. For positive γ, the most robust level of generosity is non-monotononic (as shown in **Fig. 3d**). For small crosstalk rates, the most robust response to an increase in γ is to slightly increase q. A small increase of q is often sufficient to prevent the spread of defection across the network without being too generous towards the single defector. But once the crosstalk rate has passed a certain threshold, robustness requires players to become less generous with increasing γ. In that case, a certain spread of defection can no longer be prevented, and players instead have to minimize the payoff of the defector by decreasing q.

1.3 A general invasion analysis

When a cooperative resident strategy $(1, q)$ satisfies $q < q_M$, the above results only guarantee that residents can resist invasion by ALLD. However, in the following we show that if $q < q_M$ holds, residents are in fact able to resist *all* possible mutants with a reactive strategy. To this end, suppose a single mutant employs the strategy (p_1, q_1) whereas the remaining residents use strategy (p_2, q_2). Again, we can use Eq. (S3) to calculate the payoff π_1 of the mutant, as well as the payoff π_2 of each

resident. This yields the following payoff difference

$$\pi_1 - \pi_2 = \frac{(1-\gamma)\big(c+b(N-1)\big)\cdot\big(q_1(1-p_2)-q_2(1-p_1)\big)\cdot(r_2-r_M)}{(N-1)(1-r_2)(1-r_1r_2)+\gamma\Big((1-r_1)r_2+(N-1)r_1(1-r_2)r_2\Big)}\ . \tag{S10}$$

Here, $r_1 := p_1 - q_1$ and $r_2 := p_2 - q_2$, whereas

$$r_M := \frac{b+c(N-1)}{c+b(N-1)}\cdot\frac{1}{1-\gamma}\ . \tag{S11}$$

For the resident strategy (p_2, q_2) to be resistant against invasion, we require $\pi_1 - \pi_2 \leq 0$ for all mutant strategies (p_1, q_1). We can distinguish three cases:

Case 1: $r_2 > r_M$**.** In this case, Eq. (S10) implies for every $p_2 < 1$ that the mutant strategy ALLC with $(p_1, q_1) = (1, 1)$ can invade. Hence, resident strategies can only resist invasion if $p_2 = 1$. Conversely, if $p_2 = 1$, it follows from Eq. (S10) that $\pi_1 - \pi_2 \leq 0$ for all mutants, as required.

Case 2: $r_2 < r_M$**.** Analogously to before, Eq. (S10) implies for every $q_2 > 0$ that the mutant strategy ALLD with $(p_1, q_1) = (0, 0)$ can invade. Hence, resident strategies resist invasion if and only if $q_2 = 0$.

Case 3: $r_2 = r_M$**.** In that case, Eq. (S10) immediately implies $\pi_1 = \pi_2$, irrespective of the mutant strategy.

This analysis suggests there are three different sets of strategies that can resist invasion by single mutants. The first case corresponds to the case of cooperative strategies $(1, q)$ such that $1-q > r_M$ (or, equivalently, $q < q_M$ as defined by Eq. (S6)). The second case corresponds to the case of defective strategies $(p, 0)$ such that $p < r_M$. Finally, the last case corresponds to all strategies (p, q) that satisfy the linear relationship $p - q = r_M$.

1.4 Adaptive dynamics for well-mixed populations

Based on the above static results, we can also derive a simple deterministic model to describe how the players' strategies evolve over time, the so-called adaptive dynamics of the system [5]. We consider a well-mixed population of size N. The population is monomorphic and applies the resident strategy (p_2, q_2). This population is then invaded by a single mutant with strategy (p_1, q_1). We define the mutant's invasion fitness as $F := \pi_1 - \pi_2$, as given by Eq. (S10). Adaptive dynamics posits that evolutionary trajectories point towards the mutant with the highest invasion fitness,

$$\dot{p} = \frac{\partial F}{\partial p_1}\bigg|_{p_1=p_2=:p,\ q_1=q_2=:q} \qquad \text{and} \qquad \dot{q} = \frac{\partial F}{\partial q_1}\bigg|_{p_1=p_2=:p,\ q_1=q_2=:q}\ . \tag{S12}$$

Plugging Eq. (S10) into Eq. (S12) yields the following two-dimensional dynamical system,

$$\dot{p} = \frac{q}{1-r} \cdot h(r) \quad \text{and} \quad \dot{q} = \frac{1-p}{1-r} \cdot h(r), \tag{S13}$$

where

$$h(r) = \frac{(1-\gamma)r\big(c + b(N-1)\big) - \big(b + c(N-1)\big)}{(N-1)(1-r^2) + r\big(1+(N-1)r\big)\gamma} \ . \tag{S14}$$

Eq. (S13) implies that $\dot{p}$ and $\dot{q}$ always have the same sign. Since $h(r) = 0$ if and only if $r = r_M$, with r_M as defined in Eq. (S11), we obtain the analogous three cases as in the previous section. For initial populations (p, q) with $p - q > r_M$, both $\dot{p}$ and $\dot{q}$ are positive, and populations evolve towards higher cooperation probabilities. The 2-dimensional area in the (p, q)-space for which $p - q > r_M$ is thus called the *cooperation rewarding zone* [6]. In contrast, for initial populations with $p - q < r_M$, both $\dot{p}$ and $\dot{q}$ are negative, and we speak of the *defection rewarding zone*.

Provided that $r_M < 1$, the system is thus bistable (**Supplementary Fig. 9** shows phase portraits for two different crosstalk rates). Orbits either converge to a fully cooperative population $(1, q)$ with $q < 1 - r_M$, to a fully defective population $(p, 0)$ with $p < r_M$, or to the line of interior singular points $p - q = r_M$. In the limiting case of no crosstalk (**Supplementary Fig. 9a**) this recovers previous results on the adaptive dynamics of reciprocity in finite populations [7]. However, as the crosstalk rate γ increases, it follows from Eq. (S11) that r_M increases. Geometrically, this means that the line of fixed points is shifted to the right and the cooperation rewarding zone shrinks (**Supplementary Fig. 9b**). As γ exceeds the value of γ^* as defined by Eq. (S8), this zone vanishes altogether. Higher rates of crosstalk thus impede the evolution of cooperation, as they diminish the set of initial population that converge towards fully cooperative states.

The above results consider evolution as a deterministic process: if the initial population is in the defection rewarding zone, then the population will not employ a cooperative strategy $(1, q)$ in subsequent generations. In the main text, we have thus contrasted this deterministic model of adaptive dynamics with a stochastic imitation process. According to the imitation process, an ALLD population may be invaded by a cooperative mutant, even if this mutant is initially at a disadvantage in a population of defectors. To explore which mutants are particularly likely to invade, **Supplementary Fig. 7** represents the success of all possible mutant strategies if the resident population either employs a noisy variant of ALLD, (0.001, 0.001), or a noisy variant of GTFT, (0.999, 0.333). When crosstalk is rare, ALLD populations are less robust against mutant invasions. On average, it only takes 41 successive mutant invasions until the first mutant fixes. Moreover, successful mutants typically exhibit TFT-like behavior, and are thus often in the cooperation-rewarding zone. In contrast, if residents employ GTFT, it takes 697 mutants until the first mutant fixes, and successful mutants are highly cooperative themselves. However, ALLD outperforms GTFT when crosstalk is frequent. For $\gamma = 0.5$, we observe that it typically takes 116 mutants until an ALLD population is successfully replaced (again, TFT-like strategies are most likely to fix in an ALLD population).

In contrast, GTFT is already invaded after 105 mutant strategies, and successful mutants are no longer similar to GTFT. These simulation results again highlight that high crosstalk rates undermine the evolutionary robustness of cooperation. For large values of γ, cooperative strategies become unstable, and defective strategies prevail.

1.5 The evolutionary relevance of extortionate strategies under crosstalk

When the population only consists of $N = 2$ individuals with respective strategies (p_1, q_1) and (p_2, q_2), their respective payoffs according to Eq. (S3) become

$$\begin{aligned} \pi_1 &= \frac{(q_2 + q_1 r_2)b - (q_1 + q_2 r_1)c}{1 - r_1 r_2}, \\ \pi_2 &= \frac{(q_1 + q_2 r_1)b - (q_2 + q_1 r_2)c}{1 - r_1 r_2} \;. \end{aligned} \tag{S15}$$

These two payoffs satisfy the linear relationship

$$\pi_1 = \frac{br_2 - c}{b - cr_2} \cdot \pi_2 + \frac{(b-c)(b+c)q_2}{b - cr_2} \;. \tag{S16}$$

In particular, by choosing a strategy of the form $(p_2, 0)$, player 2 can enforce the relation

$$\pi_1 = \frac{bp_2 - c}{b - cp_2} \cdot \pi_2 \;. \tag{S17}$$

Since $c < b$ and $0 \le p_2 \le 1$, it follows that $\pi_2 \geq \pi_1$, irrespective of player 1's strategy (for $p_2 < 1$, equality only holds if both players get the mutual defection payoff 0). Moreover, by choosing $p_2 > c/b$, player 2 makes sure that the two payoffs π_1 and π_2 are positively related. In that case, if the co-player 1 aims to maximize her own payoff, she automatically maximizes player 2's payoff as well. Strategies of the set

$$E = \Big\{ (p,q) \in [0,1]^2 \;\Big|\; c/b < p < 1,\; q = 0 \Big\} \tag{S18}$$

have thus been termed extortionate [8]. With an extortionate strategy, players can ensure that they almost always outperform the opponent. At the same time, it is in the opponent's best interest to be unconditionally cooperative.

Here, we aim to explore when such extortionate strategies are stable in populations of size N in the presence of crosstalk. Case 2 in Section 1.3 implies that extortionate strategies need to satisfy

$$\frac{c}{b} < p < \frac{b + c(N-1)}{c + b(N-1)} \cdot \frac{1}{1-\gamma} \;. \tag{S19}$$

to resist invasion by all mutant strategies. We note that this condition is automatically satisfied

if $N = 2$, recovering previous results that extortion can succeed in small populations [1, 2]. But while generic models of direct reciprocity (with $\gamma = 0$) predict that extortionate strategies become unstable in large populations, condition (S19) suggests that crosstalk can stabilize extortion. In particular, once $\gamma > \gamma^*$ (as defined in Eq. (S8)), *every* extortionate strategy $(p, 0)$ is resistant against mutant invasions. At the same time, however, it should be noted that by becoming stable, extortionate strategies lose their most appealing property when crosstalk rates are high. For high values of γ, the best response in a population of extortioners is no longer to give in and to cooperate unconditionally. The best response is to be extortionate as well.

In **Supplementary Fig. 8**, we show simulations using the stochastic imitation process considered in the main text. We consider a resident population that employs a given extortionate strategy $(p, 0) \in E$. For this resident population, we calculate the fixation probability for all possible reactive mutant strategies, as well as the average time it takes until a random mutant replaces the resident. These simulations support the above analytical findings. For moderate population sizes and no crosstalk, the extortionate strategy is quickly invaded by more cooperative strategies (**Supplementary Fig. 8a**). As the crosstalk rate γ increases, the extortionate strategy become more robust against mutant invasions, and successful mutants typically show the characteristics of extortionate strategies themselves (**Supplementary Fig. 8b**). We have also explored the evolutionary relevance of extortionate strategies by measuring how often the evolving population visits a δ-neighborhood of E. The respective fraction of time increases substantially as the crosstalk rate γ increases (**Supplementary Fig. 8c**). We conclude that under crosstalk, extortionate strategies are able to persist even in larger populations.

1.6 Stochastic evolutionary dynamics for a birth-death process

In the main text, we have considered a cultural evolution setup to describe how strategies in a population change over time. We have assumed that strategies that perform well are more likely to be imitated by other players. Similarly, we can also study the dynamics when strategies spread by inheritance, and not by imitation. To this end, let us consider a Moran process. As in the main text, we consider a population of individuals that engage in repeated games subject to crosstalk. Each individual i acts according to a fixed strategy (p_i, q_i) that is now genetically determined. The payoff π_i of individual i again is determined by Eq. (3) of the main text. This payoff translates into an individual fitness $f_i = \exp(s\pi_i)$, with $s \geq 0$ being again the strength of selection (the exponential fitness mapping ensures that the players' fitness is always positive). We assume that in each evolutionary time step one individual is chosen for reproduction (proportional to its fitness), and that its offspring replaces a randomly chosen individual. The offspring inherits the parent's strategy with probability $1 - \mu$, and it adopts a new reactive strategy with probability μ, where μ is the mutation rate. For this Moran process, the fixation probability of a single mutant in a

well-mixed population of $N-1$ residents is given by Ref. [9]:

$$\rho_F = \left(1 + \sum_{i=1}^{N-1} \prod_{j=1}^{i} \exp\left[-s\big(\pi_M(j) - \pi_R(j)\big)\right]\right)^{-1} . \tag{S20}$$

Here, $\pi_M(j)$ and $\pi_R(j)$ are the mutant's and the resident's payoff in a population with j mutants, respectively. This fixation probability coincides with the respective fixation probability for the pairwise imitation process [10]. In particular, all corresponding evolutionary results for the imitation process (**Fig. 4c,d**) considered in the main text equally apply to the Moran process discussed here.

2 Further results for arbitrary population structures

2.1 Expected recovery time after errors

So far we have been concerned with the effects of crosstalk on the stationary cooperation rates and payoffs in a population. In particular, we have seen that crosstalk can lead to a spread of defection across a network. Herein we are interested in the respective timescale. How long does it take until an isolated defection event (e.g. due to an error) is "forgotten" in a generally cooperative population?

To this end, we consider a population of size N on a regular network with degree k. All players apply the strategy $(1, q)$, and all players are in state C initially. Suppose that due to an error in the very first game, one of the players defects and that in all subsequent rounds no more errors occur and players act according to their strategies. We are interested in the recovery time, in other words, the time it takes until all players are in state C again.

In the limiting case of no crosstalk, this recovery time can be calculated analytically. Since $\gamma = 0$, an error only affects the edge between the pair of players that has interacted in the very first game. Moreover, within this pair there is always at most one player who is in the D state (because in each round, at least one of the players cooperates and players have $p = 1$). As the probability that a specific edge of the regular graph is chosen is $2/(N \cdot k)$, we can calculate the probability that the population recovers after exactly t rounds as follows:

	Probability	Explanation
**Recovery after 1 round**	q	Player 2 immediately forgives defecting player 1.
**Recovery after 2 rounds**	$(1-q)\frac{2q}{N\cdot k}$	Player 2 does not forgive immediately, but the same edge is chosen in the second round, player 2 defects and player 1 forgives player 2.
⋮	⋮	⋮
**Recovery after t rounds**	$(1-q)\left(1-\frac{2q}{N\cdot k}\right)^{t-2}\frac{2q}{N\cdot k}$	No recovery in first $t-1$ rounds, but in the t-th round the respective edge is chosen and the co-player of the defector forgives.

Therefore, the expected recovery time T_γ for $\gamma = 0$ is

$$T_0 = q + (1-q)\frac{2q}{Nk}\sum_{t=2}^{\infty} t\left(1-\frac{2q}{Nk}\right)^{t-2} = 1 + \frac{Nk(1-q)}{2q}\ . \tag{S21}$$

In particular $q = 1$ implies $T_0 = 1$. The expected recovery time T_0 has the following two properties:

1. For any given q, the recovery time is monotonically increasing in k (i.e., recovery always takes longer in the complete graph than in the circle).
2. For any given k, the recovery time is monotonically decreasing in q (i.e., recovery always occurs faster when players are more forgiving).

These two properties hold in fact for any crosstalk rate, as further simulations show (**Supplementary Fig. 6**). Moreover, these simulations also show the recovery time is a decreasing function of γ. Intuitively, under crosstalk each player's automaton is more likely to be updated during a single interaction. Given that the residents have $p = 1$ and $q > 0$, these updating events on average increase the cooperation level in a population: a player's D state is more likely to be overridden by a C than the other way around.

2.2 Crosstalk based on aggregate experience

In the so far explored models of crosstalk, a player who needs to decide whether to cooperate or not only considered single experiences (either with the present co-player, or with the previous co-player). Instead one may also consider a model where decisions are based on a player's aggregate experience in previous games. In the following, we sketch a simple model for that case.

Again, we consider a population of size N, and each player holds a two-state automaton for each of her co-players. This automaton is in state C if the respective co-player has cooperated in the previous round, and it is in state D otherwise. To encode the present state of a player's automaton at time t, we use the variable x_{ij}^t. The value of this variable is $x_{ij}^t = 1$ if in the last interaction between i and j prior to round t, player j cooperated. Otherwise, we set $x_{ij}^t = 0$. Suppose now that in round t, players i and j interact. We assume that prior to her decision which action to choose, player i considers a weighted average score across all her co-players' previous decisions,

$$\bar{x}_{ij}^t(\gamma) = (1-\gamma) \cdot x_{ij} + \gamma \sum_{k=1}^{N} \frac{w_{ik}}{\bar{w}_i} x_{ik}^t. \tag{S22}$$

As before, we interpret γ as the model's crosstalk rate. In the limiting case $\gamma = 0$, there is no crosstalk and only the direct co-player's previous action is taken into account. In the other limiting case $\gamma = 1$, there is full crosstalk and player i simply considers the average cooperation rate across all her co-players. Given the average score $\bar{x}_{ij}^t(\gamma)$, we assume that player i with strategy (p_i, q_i) cooperates with probability p_i if $\bar{x}_{ij}^t(\gamma) \geq \tau$, and otherwise cooperates with probability q_i. The parameter τ_i denotes an exogenous cooperation threshold. In the special case $\gamma = 0$, the above model is equivalent to the standard model of reactive strategies of direct reciprocity [3, 6] for any $0 < \tau < 1$. However, for positive values of γ, players do not only respond to their direct co-player, but they are also affected by outside experiences with previous co-players.

First, we explore the above model using computer simulations. To this end, we consider players using the strategy $(1, 1/3, \tau)$, to which we refer to as *Aggregate Generous Tit-for-Tat* (AGTFT). For four different population structures (cycle, lattice, 6-regular graph and complete graph), we study the cooperation dynamics that arise in a population in which one player applies ALLD and all other players apply AGTFT. In **Supplementary Fig. 11**, we show the resulting payoffs for three different values of the cooperation threshold $\tau \in \{0.2, 0.5, 0.8\}$ and for different crosstalk rates $0 \leq \gamma \leq 1$. As expected, in all population structures the GTFT players gain a higher payoff than the ALLD player in the absence of crosstalk. However, as the crosstalk rate increases, the ranking of strategies can change once γ exceeds a certain threshold. There are two qualitative changes that can occur. When τ is relatively low compared to γ, AGTFT players start to fully cooperate with the ALLD player (in **Supplementary Fig. 11**, the red curve jumps from $\pi_D = 1$ to $\pi_D = 3$). On the other hand, when τ is high compared to γ, already a single defector in the population can prevent AGTFT players to fully cooperate with other AGTFT players (in **Supplementary Fig. 11a,b**, this happens when the blue curve for $\tau = 0.8$ jumps from $\pi_A \approx 2$ to $\pi_A < 1$).

To understand these discontinuous transitions in the players' payoffs, we calculated when *AGTFT* players fully cooperate among themselves, and when they fully cooperate with the ALLD player. This yields three different cases:

1. *AGTFT players fully cooperate with everyone.* This case applies if the average cooperation

rate $\bar{x}_{ij}^t$ is always above the threshold τ, even if the respective co-player is a defector. By Eq. (S22), this yields the condition

$$\gamma \cdot \frac{N-2}{N-1} \geq \tau \ . \tag{S23}$$

2. *AGTFT players are fully cooperative among themselves, but they only cooperate against the defector with probability q.* This case applies if $\bar{x}_{ij}^t \geq \tau$ in case the co-player uses AGTFT, whereas $\bar{x}_{ij}^t < \tau$ if the co-player used ALLD. By Eq. (S22), this yields the following condition,

$$\gamma \cdot \frac{N-2}{N-1} < \tau \leq (1-\gamma) + \gamma \frac{N-2}{N-1} \ . \tag{S24}$$

3. *AGTFT players are no longer fully cooperative among themselves.* This case applies if $\bar{x}_{ij}^t < \tau$ even if the co-player adopts AGTFT and even if all AGTFT players have cooperated in the previous round. This yields

$$(1-\gamma) + \gamma \frac{N-2}{N-1} < \tau \ . \tag{S25}$$

In **Supplementary Fig. 12**, we show the parameter regions (γ, τ) that satisfy the three inequalities (S23), S24, and (S25). For all population structures, we find that if τ is too small, the AGTFT population cooperates with everyone. On the other hand, if τ is too large, AGTFT do not even fully cooperate among themselves. Only when τ is intermediate, the AGTFT players succeed in keeping the defector's payoff low, while still maintaining full cooperation among themselves. Surprisingly, we find that this region in the (γ, τ)-space always has an area of 1/2. Independent of the population structure, half of the parameter combinations are amenable to AGTFT to be stable against defectors. However, as in our original model, we find that, all other parameters kept constant, it is easier for AGTFT to succeed against ALLD if γ is small (**Supplementary Fig. 12**).

Supplementary References

[1] C. Hilbe, M. A. Nowak, and K. Sigmund. The evolution of extortion in iterated prisoner's dilemma games. *Proceedings of the National Academy of Sciences USA*, 110:6913–6918, 2013.

[2] A. J. Stewart and J. B. Plotkin. From extortion to generosity, evolution in the iterated prisoner's dilemma. *Proceedings of the National Academy of Sciences USA*, 110(38):15348–15353, 2013.

[3] M. A. Nowak and K. Sigmund. Tit for tat in heterogeneous populations. *Nature*, 355(6357):250–253, 1992.

[4] P. Molander. The optimal level of generosity in a selfish, uncertain environment. *Journal of Conflict Resolution*, 29:611–618, 1985.

[5] J. Hofbauer and K. Sigmund. *Evolutionary Games and Population Dynamics.* Cambridge University Press, Cambridge, UK, 1998.

[6] K. Sigmund. *The Calculus of Selfishness.* Princeton Univ. Press, 2010.

[7] L. A. Imhof and M. A. Nowak. Stochastic evolutionary dynamics of direct reciprocity. *Proceedings of the Royal Society B*, 277:463–468, 2010.

[8] W. H. Press and F. D. Dyson. Iterated prisoner's dilemma contains strategies that dominate any evolutionary opponent. *Proceedings of the National Academy of Sciences USA*, 109:10409–10413, 2012.

[9] A. Traulsen and C. Hauert. Stochastic evolutionary game dynamics. In Heinz Georg Schuster, editor, *Reviews of Nonlinear Dynamics and Complexity*, volume II, pages 25–61. Wiley-VCH, Weinheim, 2009.

[10] Bin Wu, Benedikt Bauer, Tobias Galla, and Arne Traulsen. Fitness-based models and pairwise comparison models of evolutionary games are typically different—even in unstructured populations. *New Journal of Physics*, 17:023043, 2015.
